# An expandable, modular de novo protein platform for precision redox engineering

**DOI:** 10.1073/pnas.2306046120

**Published:** 2023-07-24

**Authors:** George H. Hutchins, Claire E. M. Noble, H. Adrian Bunzel, Christopher Williams, Paulina Dubiel, Sathish K. N. Yadav, Paul M. Molinaro, Rob Barringer, Hector Blackburn, Benjamin J. Hardy, Alice E. Parnell, Charles Landau, Paul R. Race, Thomas A. A. Oliver, Ronald L. Koder, Matthew P. Crump, Christiane Schaffitzel, A. Sofia F. Oliveira, Adrian J. Mulholland, J. L. Ross Anderson

**Affiliations:** ^a^School of Biochemistry, University of Bristol, University Walk, Bristol BS8 1TD, United Kingdom; ^b^BrisSynBio Synthetic Biology Research Centre, Life Sciences Building, University of Bristol, Bristol BS8 1TQ, United Kingdom; ^c^School of Chemistry, University of Bristol, Bristol BS8 1TS, United Kingdom; ^d^Department of Physics, The City College of New York, New York, NY 10031; ^e^Graduate Programs of Physics, Biology, Chemistry and Biochemistry, The Graduate Center of The City University of New York, New York, NY 10016

**Keywords:** protein design, bioenergetics, heme proteins

## Abstract

The electron-conducting circuitry of life represents an as-yet untapped resource of exquisite, nanoscale biomolecular engineering. Here, we report the characterization and structure of a de novo diheme “maquette” protein, 4D2, which we subsequently use to create an expanded, modular platform for heme protein design. A well-folded monoheme variant was created by computational redesign, which was then utilized for the experimental validation of continuum electrostatic redox potential calculations. This demonstrates how fundamental biophysical properties can be predicted and fine-tuned. 4D2 was then extended into a tetraheme helical bundle, representing a 7 nm molecular wire. Despite a molecular weight of only 24 kDa, electron cryomicroscopy illustrated a remarkable level of detail, indicating the positioning of the secondary structure and the heme cofactors. This robust, expressible, highly thermostable and readily designable modular platform presents a valuable resource for redox protein design and the future construction of artificial electron-conducting circuitry.

## Significance

The flow of electrons within protein-based circuitry is essential to life, underpinning cellular energy generation and photosynthesis. While our understanding of this natural electron-conducting machinery has benefitted from the advances of the structural genomics era, we have yet to fully exploit the exceptional features of these bioelectronic components and assemblies on our own terms. To directly address this, we report the design of an expandable, modular protein platform for creating well-folded, new-to-nature proteins containing one or more redox-active heme cofactors. We also demonstrate that a relatively simple computational design strategy can be used to extend heme-containing modules into a 7-nm molecular wire and how fundamental biophysical properties of hemes within such proteins can be predicted and manipulated using computation.

Computational, de novo protein design has attained a level of sophistication where atomistic precision is almost routine (1, 2), and there now exists a multitude of examples demonstrating command over these fundamental biomolecular building blocks (3, 4). Conversely, the precision de novo–design of proteins that bind ligands, and especially redox-active cofactors, remains a challenge (5, 6). For instance, where oxidoreductase cofactors have been successfully incorporated into simple, de novo protein scaffolds (7–11), principally termed maquettes, there are few examples where high-resolution structural information has been successfully obtained (12–17). This shortfall hinders the downstream engineering of these robust and versatile scaffolds to incorporate substrate binding sites, tailor active site residues, and fine-tune cofactor biophysical properties in a predictable manner. Ultimately, such exquisite control of structure will lead to significant improvements in these proteins, aiding the expansion of their functional and catalytic repertoire, and enabling, for instance, imprinted regio- and stereoselectivity in de novo oxidoreductase enzymes.

Despite the drive toward well-packed, native-like states in de novo proteins, it should be noted that certain heme-containing maquettes exhibit catalytic activities comparable to their natural counterparts while adopting conformationally dynamic structures more reminiscent of molten globules than well-folded native-like states (18–20). In these cases, the dynamic nature of the protein may in fact enhance catalytic activity by lowering barriers to substrate entry and product exit (18, 21). However, the relationship between dynamics and catalysis in these simple proteins currently remains unclear (18, 22, 23). Since these activities extend to industrially and biosynthetically valuable reactions, it would be prudent to explore this relationship in greater depth and establish a framework of robust, engineerable de novo proteins to address these and other fundamentally important questions relating to biologically relevant phenomena, such as electron transfer.

To these ends, we describe here the design and construction of single and multiheme proteins with well-defined structures and biophysical properties that can be predictably fine-tuned. Our strategy was based on the successful design and characterization of the D2 peptide by Ghirlanda et al. (24), which self-assembles into a diheme tetrahelical bundle. Subsequent work on similar parameterized designs with D2 symmetry demonstrated that tetrahelical peptide assemblies of varying lengths could be designed in a modular fashion and with the capability of binding up to four nonbiological iron porphyrins with high specificity (10, 11). However, these pioneering, early studies principally relied on synthetic methodologies and in vitro peptide self-assembly in the presence of the selected porphyrins, and while some designs exhibited promising 1D or 2D NMR spectra (10, 24), no high-resolution structural data were reported. In contrast, we wished to create robust, expressible designs with a nativelike structure that preferably bound natural tetrapyrrole cofactors (e.g., heme B) with high affinity in vivo. To this end, we created a single-chain variant of the D2 peptide with nanomolar heme affinity, 4D2, that we were able to crystalize, obtaining a high-resolution structure of the heme-bound maquette. This structure guided the subsequent computational design of a rigid monoheme maquette and an extended tetraheme maquette. We obtained further structural insight into our designs using NMR spectroscopy and cryogenic electron microscopy (cryo-EM), the latter enabling heme-enhanced visualization of our 24-kDa tetraheme maquette which represents the current mass limit of the technique. This structural information and our confidence in the fidelity of the design process enabled the incorporation of continuum electrostatic calculations (25) into our design pipeline to produce further maquettes with predictably altered redox potentials. Such fine-tuning of a fundamental biophysical property of the cofactor is central to heme protein engineering and the future construction of catalytically efficient oxidoreductases.

## Results and Discussion.

### Conversion of the D2 Peptide to an In Vivo–Expressed Single-Chain Protein, 4D2.

**Fig. 1.**
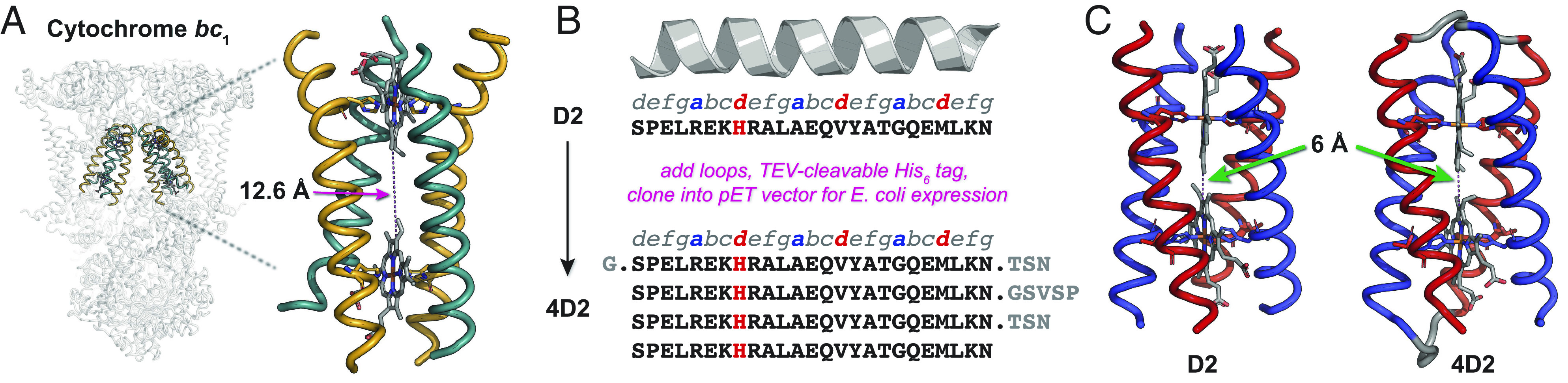
Design of 4D2. (*A*) The transmembrane diheme cytochrome *b* from the avian cytochrome *bc*_1_ complex (PDB: 1BCC) (26). The dashed line indicates the edge-to-edge distance between the conjugated tetrapyrrole ring systems of the heme B molecules. (*B*) Conversion of the D2 peptide into 4D2, incorporating two TSN loops between helices 1 and 2 and 3 and 4 and a longer GSVSP loop between helices 2 and 3. (*C*) Models of the D2:heme B assembly and 4D2, demonstrating the 6-Å edge-to-edge (between the conjugated tetrapyrrole ring systems of the hemes) distance between bound hemes, significantly shorter than the 12.6 Å present in the *bc*_1_ complex.

To enable a higher precision design process than that employed in the creation of earlier maquettes, we selected the D2 peptide (24) as a starting point for further design. D2 was originally designed by parameterizing, as a coiled coil according to the Crick parameters, the transmembrane cytochrome *b* subunit of the cytochrome *bc*_1_ complex (26) (Fig. 1). This was achieved using computational methods and the intuition of the designers, to define a sequence that would self-assemble into a soluble tetrahelical bundle with overall pseudo-D2 symmetry in the presence of heme (24). The resulting peptide demonstrated potentially high, though undefined by the authors, affinity for two heme B molecules within the assembly, and relatively well-resolved 2D ^1^H-^15^N HSQC NMR spectra were observed with two molecules of a symmetric heme B analogue (Fe protoporphyrin III) bound. Despite these promising observations, no high-resolution structural information was obtained. We reasoned that the heme B binding affinity could be improved by creating a single-chain tetrahelical bundle, preorganizing the heme-binding sites and reducing the entropic cost of assembly in the unconnected D2:heme B complex, thus increasing the affinity for heme B. Given the computational design of the heme-binding sites and NMR data, it was also hoped that the single-chain variant would retain the desirable structural characteristics of the original assembly.

To realize this single-chain variant of D2, termed 4D2, we designed a protein where four copies of the 25-residue D2 peptide were linked together by three short loops: two threonine-serine-asparagine (TSN) loops between helices 1 to 2 and 3 to 4 and one glycine-serine-valine-serine-proline (GSVSP) sequence at the central loop between helices 2 to 3 (Fig. 1 *B* and *C*). We also included a TEV (Tobacco etch virus N1A) protease-cleavable hexahistidine tag and V5 epitope at the N terminus (*SI Appendix*, Fig. S1) to enable purification by metal affinity chromatography and antibody detection, respectively, resulting in a 112-amino acid four-helix bundle after TEV-cleavage. Following creation of a synthetic gene and expression in *Escherichia coli* from pET151, a vibrantly red cell pellet was obtained, indicating high levels of 4D2 expression and of in vivo heme B loading (*SI Appendix*, Fig. S2). While cytoplasmic expression of 4D2 with or without supplementation with the heme precursor δ-aminolevulinic acid can result in complete heme loading, the reliability of in vivo cofactor incorporation could be considerably improved by translocating the protein to the periplasm, facilitated by cloning 4D2 into a modified pMal-p4x vector (pSHT) containing a cleavable, N-terminal signal sequence for periplasmic export, used previously for the expression of de novo *c*-type maquettes (18, 27). However, substoichiometric heme-loaded cytoplasmic preparations can be recovered either through titration of hemin into purified protein, or through supplementation with excess hemin following cell lysis or purification. Reconstitution of 4D2 with heme B results in protein with identical biophysical and spectroscopic properties to periplasmically expressed 4D2 or cytoplasmically expressed 4D2 with a full heme complement. To facilitate heme B binding studies and assess the biophysical characteristics of apo-4D2, we were able to remove bound heme B using acid:2-butanone extraction (27, 28), thus providing us with a de novo protein scaffold in which we can selectively add or remove heme in vivo or in vitro.

**Fig. 2.**
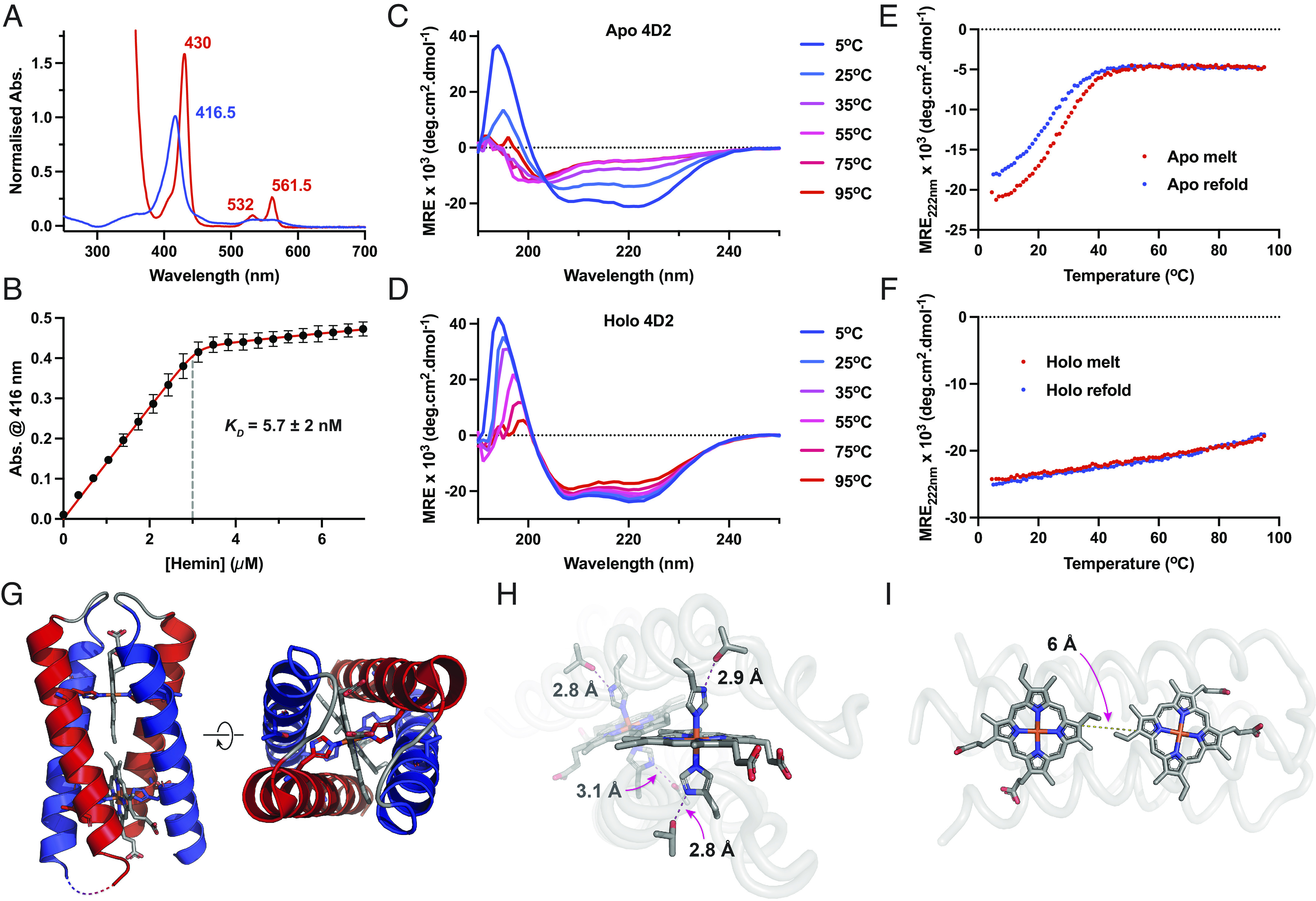
Biophysical and structural characterization of 4D2. (*A*) UV/visible absorption spectra of ferric (blue) and ferrous (red) 4D2. (*B*) Heme B binding isotherm of apo-4D2 (1.5 μM in 20 mM CHES, 100 mM KCl, pH 8.6) versus hemin in DMSO. Data were recorded in triplicate, with *error bars* representing the SD. (*C* and *D*) Far-UV circular dichroism spectra of apo-4D2 (*C*) and holo-4D2 (*D*) with varying temperature, collected in 20 mM CHES, 100 mM KCl, pH 8.6. (*E* and *F*) Temperature dependence of CD signal monitored at 222 nm during denaturation (red) and refolding (blue) for apo-4D2 (*E*) and holo-4D2 (*F*). (*G*) 1.9-Å crystal structure of 4D2, revealing the positions of the heme B molecules at the protein core, and the pseudo-D2 symmetry. (*H*) “Keystone” H-bonding interactions between the threonines and heme-coordinating histidines. (*I*) 6-Å edge-to-edge cofactor separation between hemes within 4D2 should lead to rapid intercofactor electron transfer.

Heme-bound 4D2 exhibits UV/visible absorption spectra typical of proteins binding heme B through bis-histidine ligation, with a distinctive Soret peak at 416 nm in the oxidized, ferric state (7, 29, 30) (Fig. 2*A*). We used electrospray ionization mass spectrometry under aqueous, nondenaturing conditions (31) to confirm the protein mass and further examine heme binding (*SI Appendix*, Fig. S3). Under these soft ionization conditions, we observe the diheme 4D2 complex at the predicted mass, with only a small proportion of monoheme- or apo-4D2 m/z peaks observed. Conversely, the heme groups dissociate under the conditions of MALDI mass spectrometry, resulting solely in the detection of apo-4D2. Following titrations of hemin into apo-4D2 (Fig. 2*B*), we established that 4D2 binds two molecules of heme B with high affinity, with an observed dissociation constant (*K_D_*) of <5 nM, and with no evidence of negative cooperativity. Using circular dichroism spectroscopy (CD) (Fig. 2 *C*–*F*), we also observe that heme binding dramatically increases the helicity and thermal stability of 4D2; apo-4D2 is fully unfolded at 37 °C, while diheme 4D2 demonstrates only a small loss in helicity until the start of a more cooperative melting event above 80 °C. Unlike the original D2 peptide:iron protoporphyrin III complex (24), the 2D ^1^H-^15^N HSQC spectra of diheme 4D2 exhibited only moderate signal dispersion (*SI Appendix*, Fig. S4), offering minimal potential for peak or structural assignment by NMR. This could indicate that multiple protein or side chain conformations exist and interconvert on the NMR timescale. Alternatively, another source of structural heterogeneity in diheme 4D2 could be present, perhaps as a result of the asymmetric heme B in our protein, instead of the symmetric heme analogue used by Ghirlanda et al. (24).

### Crystal Structure of a De Novo Heme-Bound Maquette.

Though the NMR indicated potential structural heterogeneity, we successfully obtained crystals of the diheme 4D2 and its single-point mutant (T19D). To elucidate the structure of 4D2, we collected datasets at four wavelengths at the I03 beamline at Diamond light source, the fluorescence profile of the two iron atoms of the heme groups facilitating experimental phase determination of 4D2 by multiwavelength anomalous dispersion (MAD) (32). We subsequently determined the crystal structures to resolutions of 1.9 Å and 2.1 Å for 4D2 and 4D2 T19D, respectively (Fig. 2*G* and *SI Appendix*, Fig. S5), using molecular replacement to elucidate the mutant structure.

The crystal structure of 4D2 matches very well to the expected fold and design, with four helices arranged in an ordered coiled coil and the four histidine residues ligating each of the two heme cofactors across opposite helices. The identical helical regions fit the pseudo-D2 symmetry of the original parameterized peptide design (24). Each histidine is contacted by a threonine within hydrogen bonding distance (2.8 to 3.1 Å) (Fig. 2*H*), most likely forming the “keystone” hydrogen bonding interactions of the original design and a common feature in natural transmembrane diheme components of respiratory enzymes (33). While the two shorter TSN loops on one side of the helical bundle are resolved in both structures, the flexible GSVSP loop between helices two and three is not observed in either (*SI Appendix*, Fig. S5). Heme plays a dominant role in the protein core, presenting a predominantly hydrophobic surface for connecting, in effect, two dimeric coiled coils with few interhelical interactions between them. The edge-to-edge distance between the conjugated tetrapyrrole rings of the heme cofactors is small (5.6 Å) (Fig. 2*I*), and they are essentially within van der Waals contact; we would predict very rapid electron transfer between the heme groups (~10^10^ s^−1^) (34) even with modest driving force (ΔG = 0.06 eV, λ = 0.7), similar to that observed in natural multiheme proteins (35).

Interestingly, there is evidence of disorder in the heme B orientations within the binding pockets, with similar electron density visible in positions 1, 2, 3, and 4 of the tetrapyrrole ring (*SI Appendix*, Fig. S6). We attribute this to the presence of two binding modes, related by a 180° rotation of the asymmetric heme, placing the 2 and 4 vinyl groups in the apparent 1 and 3 methyl positions of the corresponding other orientation. However, there is little evidence of significant structural rearrangement as a result of the differing steric requirements of the heme-binding modes. This has been observed in other heme B binding proteins, including neuroglobin (36) and several bacterioferritins (37, 38), where there can be near equal occupancy of the two binding modes but with little effect on the surrounding protein. We believe that these observations may explain the poor signal dispersion in the 4D2 ^1^H-^15^N HSQC NMR spectra; combined with the repetitive sequence of the four helices, the four possible combinations of heme orientations would lead to significant peak broadening and relatively poor signal dispersion, further compounded by the paramagnetic signal broadening of the two hemes. So, while structural heterogeneity seems apparent from the NMR data, it may not be due to the global conformational dynamics of the protein, and the diheme 4D2 may indeed adopt a native-like structure in solution but with a distribution of heme orientations across the population.

### Conversion of 4D2 into a Modular Monoheme Protein.

**Fig. 3.**
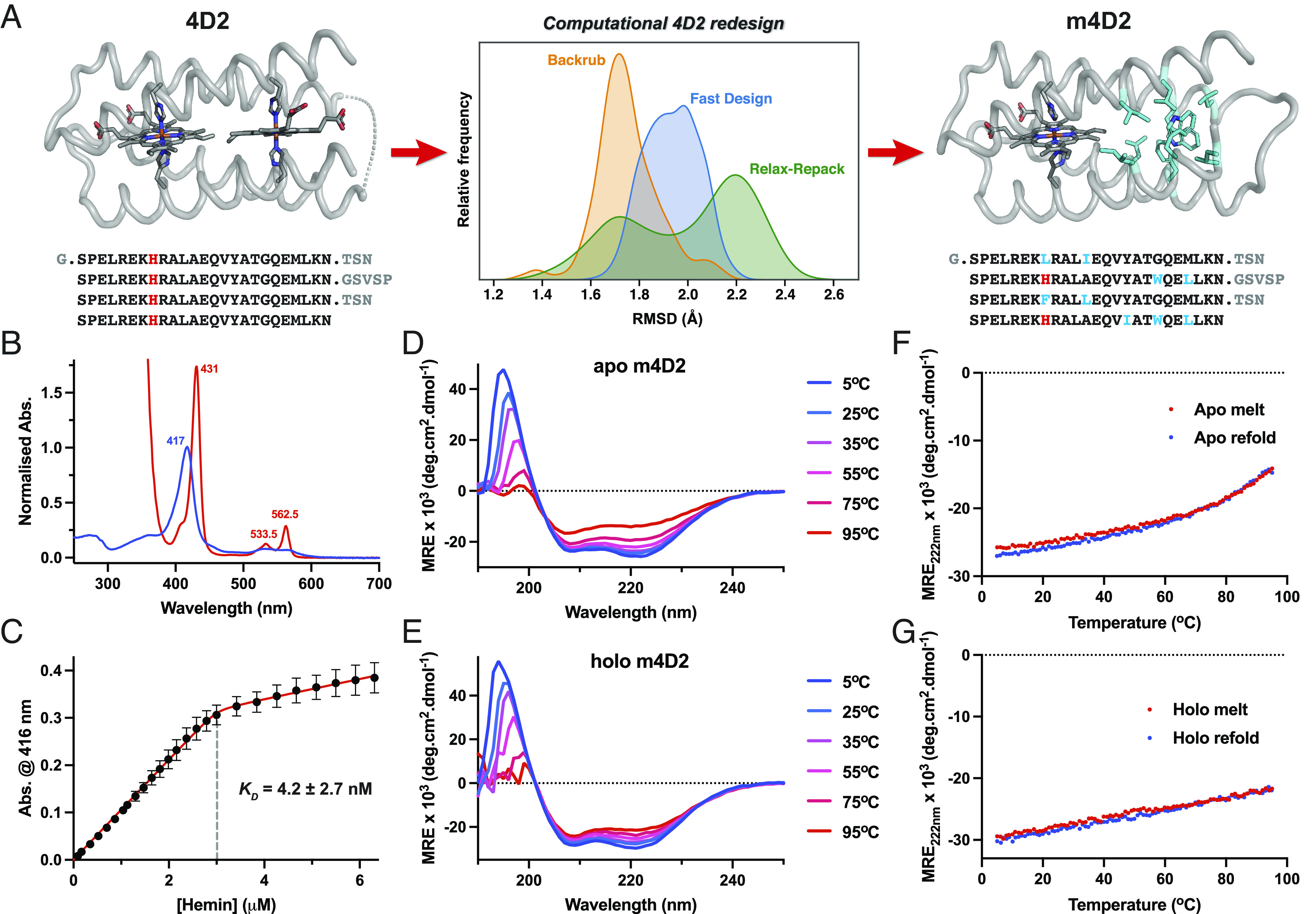
Design and biophysical characterization of m4D2. (*A*) Computational redesign of the 4D2 core using Rosetta, comparing RMSD histograms of the Backrub, Fast Design, and Relax-Repack protocols (center). Each trace represents a histogram of backbone RMSDs from 3 × 100 ns MD simulations of a single output sequence from Backrub, Fast Design, and Relax Repack protocols. These include C, N, and O atoms from all residues except those in the most flexible regions of the protein (i.e., at the *N* and *C* termini and around the GVSVP loop). (*B*) UV/visible absorption spectra of ferric (blue) and ferrous (red) 4D2. (*C*) Heme B binding isotherm of apo-m4D2 (3 μM in 20 mM CHES, 100 mM KCl, pH 8.6) versus hemin in DMSO. Data were recorded in triplicate, with *error bars* representing the SD. (*D* and *E*) Far-UV circular dichroism spectra of apo-m4D2 (*D*) and holo-m4D2 (*E*) with varying temperature, collected in 20 mM CHES, 100 mM KCl, pH 8.6. (*F* and *G*) Temperature dependence of CD signal monitored at 222 nm during denaturation (red) and refolding (blue) for apo-m4D2 (*E*) and holo-m4D2 (*F*).

Following the successful determination of the 4D2 structure, we reasoned that the scaffold could provide a template for further heme protein design, enabling us to access single and multiheme proteins with similar atomistic control. We initially designed a monoheme variant, m4D2, using Rosetta (39) to remove one of the heme-binding sites and repack the vacated binding pocket in the protein core (Fig. 3*A*), effectively splitting the protein into separate and equally sized heme-binding and packing modules. We selected the heme-binding site adjacent to the termini and longer GSVSP loop for removal as we observed, and wished to maintain, a favorable hydrogen bonding network between a single heme propionate at the other binding site and the two asparagine side chains (N29 & N87) from the structured TSN loops. We then employed a flexible backbone design protocol (40) to mutate key positions in the core to hydrophobic amino acids. To minimize unnecessary and potentially destabilizing changes to the protein, we used SOCKET (41) to identify key knobs-into-holes interactions and avoided modification of the residues involved in these contacts. In total, we selected 11 residues for the redesign process, representing about 10% of the total protein, of which, 9 were mutated in the final sequence of m4D2. The flexible backbone protocol we employed utilized the backrub method (42) for backbone sampling (*SI Appendix*, Fig. S7), applying relatively minor changes to the structure relative to the initial crystal structure. To achieve this, we adapted a Rosetta script created by Pollizi (43), used for the design of a photoactive porphyrin-binding tetrahelical bundle, generating 50 unique protein sequences. More aggressive backbone sampling methods such as the FastDesign mover or alternating rounds of sequence design (FixBB) and FastRelax were also tested, again, generating 50 unique sequences in each case; while these methods converged to a lower overall Rosetta energy score, molecular dynamics (MD) simulations of the Rosetta-output apo-m4D2 design suggested that these approaches caused significant disruption to the overall structure (*SI Appendix*, Fig. S7 *A*–*F*). MD simulations of the holo-m4D2 created using the Backrub method demonstrated minimal deviation from the Rosetta-output structure, indicating that this design was suitable for expression and characterization (*SI Appendix*, Fig. S7*G*).

After cloning the monoheme m4D2 into the same expression vector as 4D2, we expressed the protein in the *E. coli* cytoplasm using the same method as for 4D2. Like 4D2, m4D2 binds heme B in vivo and retains it through purification, resulting in a ferric UV/visible absorption spectrum almost indistinguishable from 4D2 and similarly high heme-binding affinity (*K_D_* = 4.2 nM) (Fig. 3 *B* and *C*). To confirm the heme-binding stoichiometry of m4D2, we performed nondenaturing mass spectrometry on the design, revealing the intended 1:1 heme-bound complex (*SI Appendix*, Fig. S3). We subsequently employed CD spectroscopy to probe m4D2 secondary structure and thermal stability (Fig. 3 *D*–*G*). We observed a predictably high degree of helicity for heme-loaded m4D2, though in contrast to 4D2, apo-m4D2 exhibits a relatively high degree of helicity and good thermal stability, with a cooperative melt transition beginning at approximately 60 °C. These observations are consistent with our design methodology and that previously implemented by Polizzi (43), where the packing module was designed to be well-folded while the unoccupied porphyrin binding site was simultaneously allowed to retain flexibility and enable facile heme binding.

### NMR Analysis of the m4D2 Fe(III) DMDPIX Complex.

**Fig. 4.**
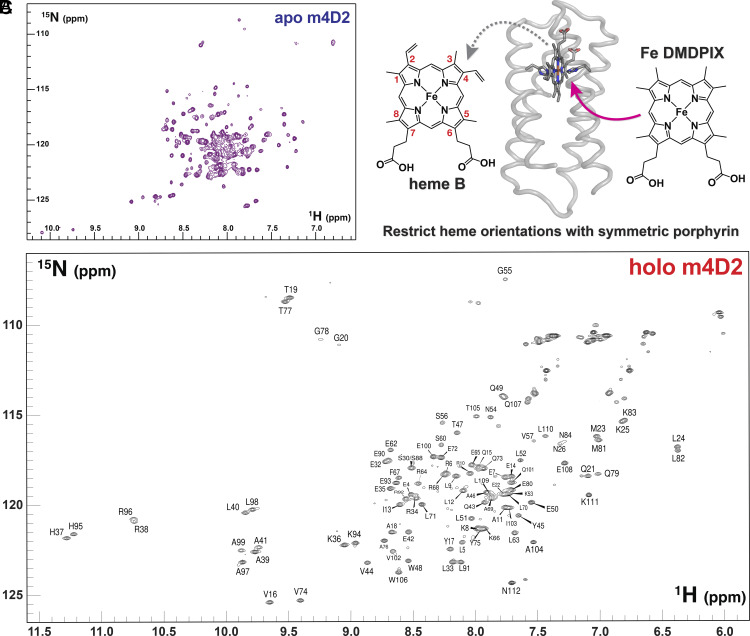
2D NMR spectroscopy of m4D2. (*A*) 700-MHz 2D ^15^N-^1^H TROSY spectrum of apo-m4D2. (*B*) Substitution of heme B for the synthetic, symmetric iron(III) 2,4-dimethyldeuteroporphyrin IX [Fe(III) DMDPIX]. Heme substituent numbers are indicated in red. (*C*) Assigned 2D ^15^N-^1^H TROSY spectrum of Fe(III) DMDPIX:m4D2 acquired at 25 ℃ and 800 MHz, demonstrating excellent signal dispersion indicative of a singular, native structure.

Unfortunately, we have thus far been unable to obtain diffraction-quality crystals of holo-m4D2. However, NMR spectroscopy demonstrated that the monoheme m4D2 was well structured, with good peak dispersion in the 2D ^1^H-^15^N HSQC spectrum (*SI Appendix*, Fig. S8), even in the absence of heme (Fig. 4*A*), validating the design strategy described above. Given the observation of alternative heme orientations in the crystal structure of 4D2, we reasoned that substitution of heme B for a symmetrical variant would eliminate such binding heterogeneity and improve NMR signal dispersion. We therefore selected the symmetric heme derivative, iron (III) 2,4-dimethyl-deuteroporphyrin [Fe(III) DMDPIX] (44) for incorporation (Fig. 4*B*), as it contains methyl groups in the 1-4 porphyrin substituent positions. Fe(III) DMDPIX binds to m4D2 with slightly lower affinity than heme B [*K_D_* = 25 nM for Fe(III) DMDPIX vs. 4.2 nM for heme B] (*SI Appendix*, Fig. S9), most likely due to the removal of hydrophobic interactions between binding site residues and the vinyl groups of heme B. With Fe(III) DMDPIX bound to double isotopically labeled m4D2 (^13^C-^15^N), we were able to obtain 3D NMR spectra that enabled the assignment of >90% of backbone and a large proportion of side chain atoms (Fig. 4*C*), demonstrating that m4D2 adopts a well-folded native-like state while paving the way for future structure determination by NMR. Of particular note are the striking chemical shift dispersions of the iron-ligating H37/H95 pair, the propionate interacting R38/R96 pair, and other hydrophobic residues clustered around the Fe(III) DMDPIX binding site (L40/98; A39/41/97/99; V16/74). These are a consequence of each amino acid’s proximity to the paramagnetic Fe(III) DMDPIX and provide excellent evidence of singular structure within the holoprotein porphyrin binding site.

Given our success with improving signal dispersion using the symmetrical heme analogue, we recorded a 2D ^1^H-^15^N HSQC spectrum of 4D2 with Fe(III) DMDPIX bound and observed a similar improvement in peak dispersion relative to heme B-bound 4D2 (*SI Appendix*, Fig. S4), further highlighting that heme-binding site heterogeneity is likely the frustrating factor in obtaining NMR spectra with signal dispersion indicative of a native state.

### Expansion of 4D2 into a Nanoscale Molecular Wire.

**Fig. 5.**
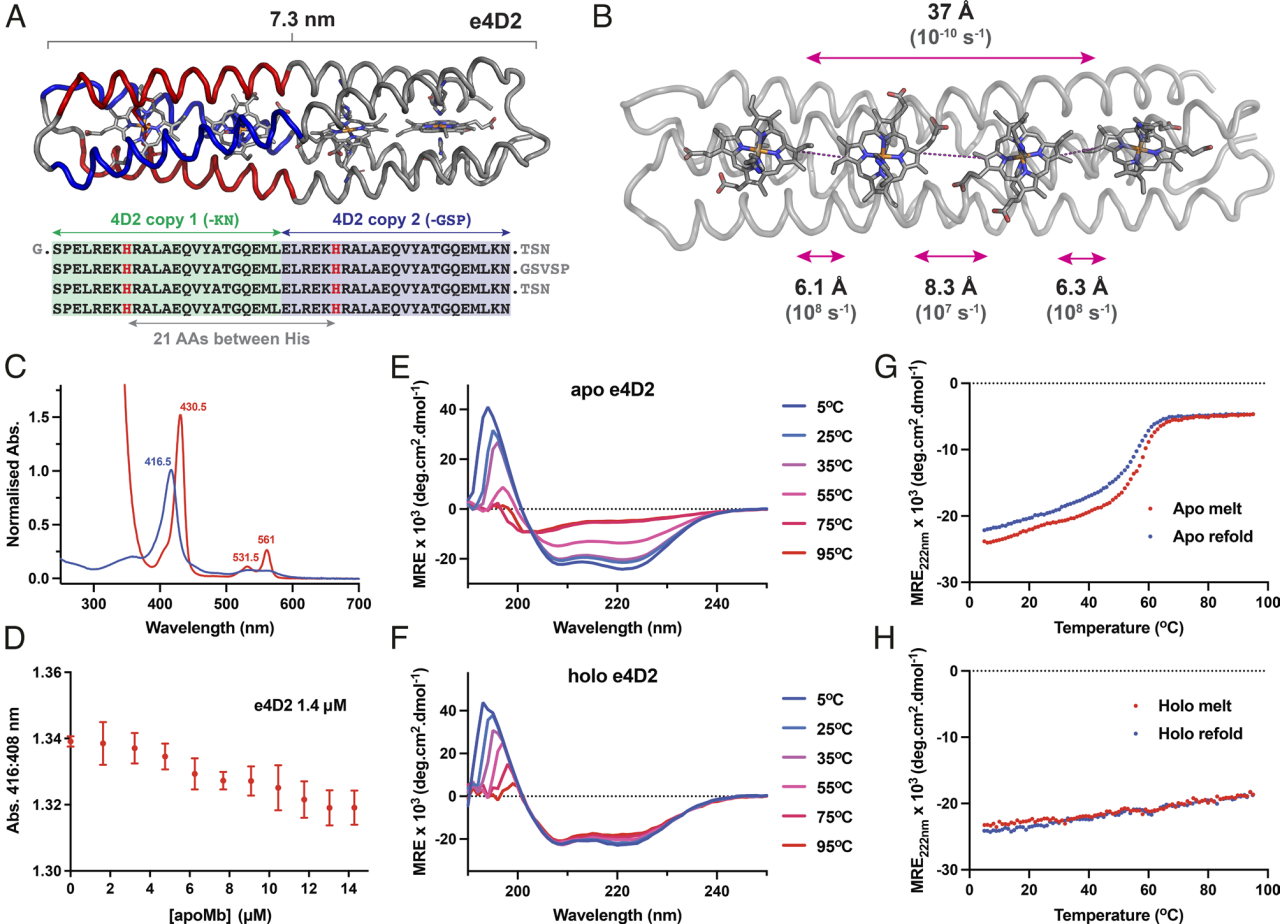
Design and biophysical characterization of e4D2. (*A*) Design strategy for e4D2, splicing two molecules of 4D2 into a longer, tetraheme B molecular wire. (*B*) Edge-to-edge electron transfer distances between cofactors in e4D2, with indicative electron transfer rates in parentheses (calculated with ΔG = 0.06 eV, λ = 0.7) (34). (*C*) UV/visible absorption spectra of ferric (blue) and ferrous (red) 4D2. (*D*) Negligible heme B transfer between holo-e4D2 and apo-myoglobin (20 mM CHES, 100 mM KCl, pH 8.6) indicates high-affinity binding of heme B to e4D2. (*E* and *F*) Far-UV circular dichroism spectra of apo-e4D2 (*E*) and holo-e4D2 (*F*) with varying temperature, collected in 20 mM CHES, 100 mM KCl, pH 8.6. (*G* and *H*) Temperature dependence of CD signal monitored at 222 nm during denaturation (red) and refolding (blue) for apo-e4D2 (*G*) and holo-e4D2 (*H*).

To facilitate the creation of nanoscale protein wires for long-range electron transfer, we designed an extended 4D2 variant, e4D2 (Fig. 5*A*), capable of binding four heme B molecules in a linear array stretching nearly 55 Å from the start to the end of the conjugated tetrapyrrole chain (Fig. 5*B*), and 73 Å from one end of the protein to the other. This required duplication of the diheme 4D2 unit, extending the protein along its helical axis, thus using a similar strategy that proved successful in the design of synthetic, tetrameric peptides that bound four nonbiological iron porphyrins (10, 11). To ensure appropriate orientation of the heme-ligating histidine side chains into the core of the protein, we separated equivalent histidine residues by 21 residues to fit three cycles of the helical heptad repeat of the 4D2 coiled-coil structure (Fig. 5*A*). We built each helix by repeating the sequence of the 25-residue 4D2 helix, removing two residues from each sequence at the junction between repeats, ensuring the correct histidine orientation. This resulted in the 46-residue e4D2 helix, for which we constructed a model using the ISAMBARD (45) design package. We extracted coiled-coiled parameters from the 4D2 crystal structure and used them to construct the extended helical structure. We then selected the same set of loops from the initial 4D2 design to link the e4D2 helices, refined and energy minimized the model using Rosetta (39), and finally ran MD simulations (*SI Appendix*, Fig. S7*H*), establishing that e4D2 was a suitable, stable scaffold for experimental characterization.

We then cloned e4D2 into the same cytoplasmic *E. coli* expression vector as 4D2 and m4D2. In contrast to 4D2 and m4D2, e4D2 does not bind a significant quantity of heme B in vivo under cytoplasmic expression, and we instead primarily purified apoprotein; however, apo-e4D2 readily and rapidly binds exogenous heme B in vitro, exhibiting a similar ferric UV/visible spectrum to both 4D2 and m4D2 (Fig. 5*C*). During the last stage of purification, the size exclusion chromatography (SEC) revealed the presence of some aggregated heme-containing protein, but also a significant quantity of heme-loaded e4D2 eluting at a volume corresponding well to that of a monomeric 25 kDa protein (*SI Appendix*, Fig. S10*A*). We found that the yield of monomeric, heme-loaded e4D2 can be improved by adding heme at 37 °C under relatively high dilution, with marked suppression of aggregated or misfolded material relative to additions at 4 and 25 °C. Once separated, this monomeric, heme-bound e4D2 remains stable for several weeks at 4 °C, and further SEC indicated only monomeric e4D2 was present (*SI Appendix*, Fig. S10*B*). Given the tendency of e4D2 to produce misfolded protein on the addition of hemin, quantification of the heme-binding affinity is challenging; however, competition assays using apo horse heart myoglobin (46) indicate that binding is tight and likely in the nanomolar range (Fig. 5*D*). To confirm the heme-binding stoichiometry of e4D2, we performed nondenaturing mass spectrometry, revealing the intended 4:1 heme-bound e4D2 complex was the dominant species present (*SI Appendix*, Fig. S3). As for the other 4D2 family proteins, we subsequently employed CD spectroscopy to probe e4D2 secondary structure and thermal stability (Fig. 5 *E*–*H*), observing a predominantly helical protein signal with excellent thermal stability in the holo, heme B-loaded form. In contrast, apo-e4D2 exhibits lower thermal stability, with a cooperative unfolding transition centered at approximately 58 °C.

### Structural Insights into the 25-kDa e4D2 by Cryo-EM.

We reasoned that the linear arrangement of the four heme iron atoms in the tetraheme e4D2 might provide sufficient electron density to aid structural analysis of the protein by electron microscopy. Despite the small size of the protein (25 kDa including the heme cofactors) lying at the current limits of cryo-EM, we were able to identify e4D2 particles by negative stain transmission electron microscopy (TEM) that corresponded to the expected dimensions of the designed protein (*SI Appendix*, Fig. S11).

**Fig. 6.**
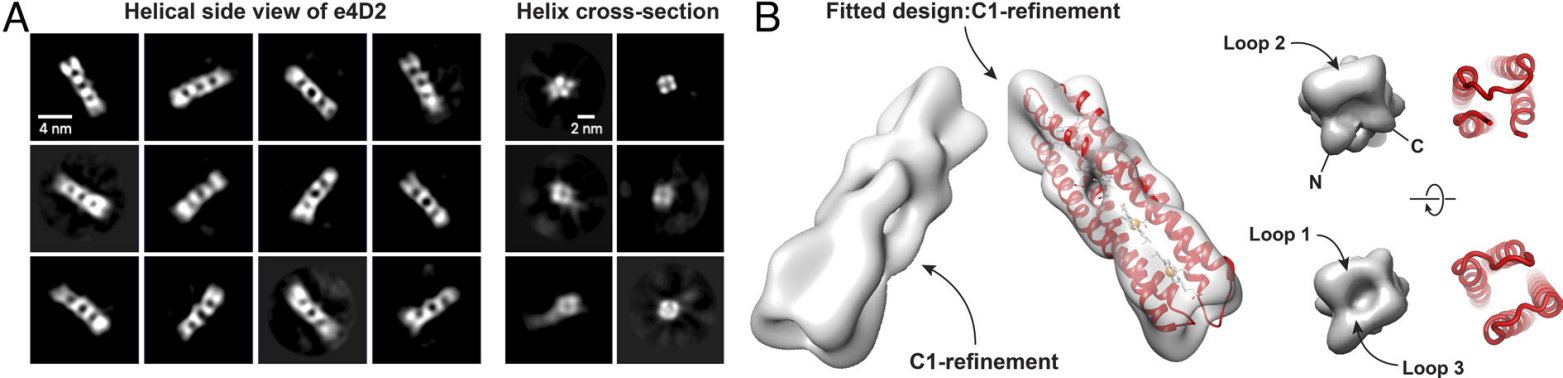
Cryo-EM analysis of the molecular wire, e4D2. (*A*) Representative, reference-free cryo-EM 2D class averages illustrate four heme-binding segments of the designed e4D2 structure (*Left*, scale bar 4 nm), while end-down class averages highlight the four-helix bundle topology (*Right*, scale bar 2 nm). (*B*) Cryo-EM reconstruction in a side (*Left*), *Top* (*Right* *Upper*), and *Bottom* (*Right* *Lower*) view, corroborating the designed topology of the e4D2 helical bundle, including the asymmetric loops at the ends of the assembly. For the side views, the structure on the left represents the cryo-EM map alone, while the right shows the e4D2 computational model fitted into the cryo-EM map.

Given these promising TEM results, we acquired a cryo-EM dataset of approximately 5,000 micrographs of e4D2, optimizing the sample grids for thin ice conditions. We then processed these images in Relion-3 (47) (*SI Appendix*, Fig. S12), identifying a set of 85,000 particles from which we generated 2D class averages and an initial 3D model. These class-averages matched well with the expected protein dimensions and showed a remarkable level of detail, demonstrating four distinct “segments” along the helical bundle which correspond to the positions of the four heme cofactors (Fig. 6*A*). Class averages representing end-down views along the helical axes also hinted at the designed four-helix bundle topology, depicting four distinct structures which we assigned as each of the four helices. We generated the initial 3D model (*SI Appendix*, Fig. S12) by refinement with D2-symmetry to maximize the available data for model-building. This symmetry is accurate for the core helical structure of the e4D2 design, though it does not consider the asymmetric connecting loops.

We further processed a subset of 13,303 particles with improved homogeneity, which we then used to refine a final 3D map without symmetry constraints (C1, *SI Appendix*, Fig. S13). This model further demonstrated the helicity of the protein, with the curvature of the helices in the Rosetta and MD-derived e4D2 model fitting well within the density map (Fig. 6*B*). Furthermore, this model highlighted the connecting loops in the protein, showing minimal density at the unconnected protein termini and symmetric density at the other end of the bundle that contains the two opposing loops. Overall, while the resolution of the cryo-EM structure is low (8.4 Å), it offers strong evidence of the designed e4D2 structure despite the exceptionally small size of the protein.

### Redox Properties of m4D2, 4D2, and e4D2.

**Fig. 7.**
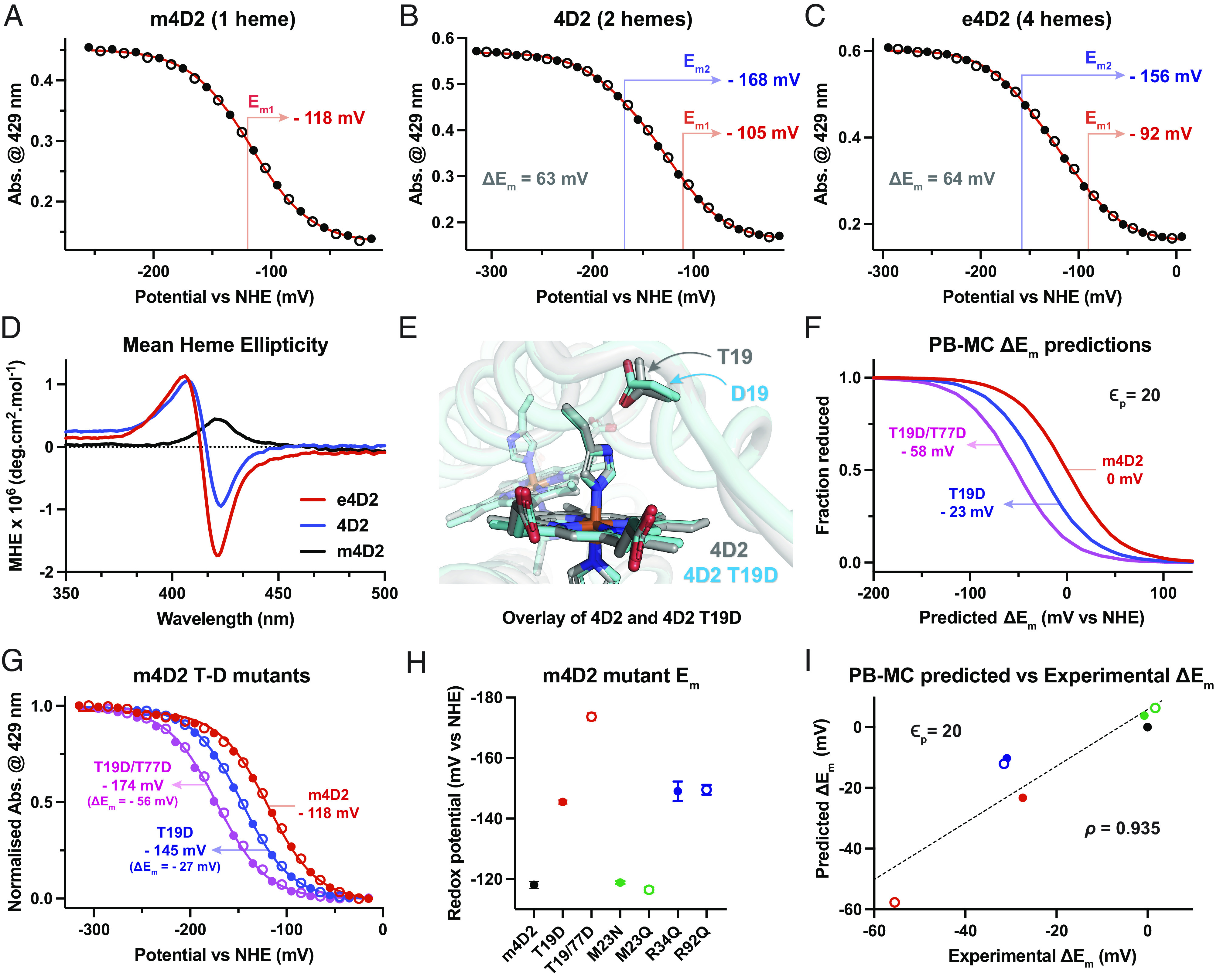
Redox characterization and engineering of the 4D2 proteins. (*A*–*C*) Redox potentiometry of m4D2 (*A*), 4D2 (*B*) and e4D2 (*C*) recorded in 20 mM CHES, 100 mM KCl, 10% glycerol, pH 8.6. Fitted potentials are indicated on the graphs, along with the split (ΔE_m_) between E_m1_ and E_m2_ for 4D2 and e4D2. (*D*) Visible circular dichroism spectra of m4D2 (black), 4D2 (blue) and e4D2 (red) reveal intercofactor exciton coupling for 4D2 and e4D2. Spectra were recorded in 20 mM CHES, 100 mM KCl, pH 8.6, and normalized first to protein and then to the heme concentrations, resulting in units of mean heme ellipticity on the *y* axis. (*E*) Structural overlay of 4D2 and 4D2 T19D demonstrating minimal structural rearrangement on mutation of the H-bonding threonine to aspartate. (*F*) PB–MC calculations of redox titrations for m4D2 (red) and its mutants T19D (blue) and T19/77D (magenta) at a dielectric constant (ε) of 20, and with the E_m_ of m4D2 set to 0 mV. (*G*) Experimentally determined redox titrations for m4D2 (red) and its mutants T19D (blue) and T19/77D (magenta), recorded in 20 mM CHES, 100 mM KCl, 10% glycerol, pH 8.6. (*H*) Measured redox potentials for m4D2 and a series of mutants. Data were recorded in triplicate, with *error bars* representing the SD. (*I*) Comparison of predicted (*y* axis) and experimentally determined (*x* axis) redox potentials for the m4D2 mutants. The PB–MC calculated values were determined at a dielectric constant (ε) of 20. The data are fit to standard linear fitting function (using a standard least squares regression), from which a correlation coefficient (ρ) of 0.935 was obtained.

Redox potential is a parameter key to the determination of electron transfer rate and direction within cofactor chains (34), while dictating the scope of catalytic activity at cofactor-centered enzyme active sites (48). To determine the heme B redox potentials in our proteins, we used optically transparent thin layer electrochemistry (49), initially measuring a potential of −118 mV vs. NHE for the monoheme m4D2 (Fig. 7*A*). For 4D2, we observed heme midpoint potentials of −105 mV and −168 mV (Fig. 7*B*), and, in notable contrast to the equivalent data obtained for the diheme D2 peptide assembly (24), there is no evidence of hysteresis in these redox titrations. The split in the 4D2 heme potentials is consistent with the expected electrostatic field effects of placing hemes in close proximity, as previously demonstrated in earlier iterations of the heme-containing maquettes (29), and the ΔE_m_ of 63 mV is comparable to those observed in diheme components of transmembrane respiratory complexes (50). Given the identical protein sequences around the two heme-binding sites, it is not apparent whether a low or high potential heme site can be assigned to those within 4D2, and while there may be a preference, further investigation is necessary to unambiguously make an assignment.

e4D2 exhibits a broadly similar potentiometric titration to 4D2, and while we initially attempted to fit the data to a Nernst function with four single electron redox processes, there were multiple solutions to differing fitting equations with near identical statistical validity. For simplicity, we decided to reduce the number of fitting parameters, and elected to use the same fitting function as for 4D2 (Fig. 7*C*). This function fit our data well, treating the four hemes as pairs with potentials of −92 and −156 mV vs. NHE. We further justified this assignment on the basis that the outer and inner hemes experience differing electric field environments as a result of their close proximity to one or two other hemes, respectively. Interestingly, the e4D2 ΔE_m_ between heme pairs (64 mV) is nearly identical to that of 4D2. There is also a progressive increase in potential for the initial heme reduced with increasing numbers of hemes in the protein, as well as an increase in both low and high potential E_m_s when moving from 4D2 to e4D2. The retention of the ΔE_m_ between 4D2 and e4D2 demonstrates that the electrostatic heme–heme coupling is maintained with the expansion of the protein to accommodate more hemes, while the general positive drift in redox potential might indicate electronic coupling between hemes within the chain, similar to that characterized and modeled in multiheme *c*-type cytochromes (51).

To further probe electronic heme-to-heme communication within our proteins, we recorded CD spectra in the visible Soret region of the heme spectrum, scaling derived molar ellipticities to the number of hemes within our proteins (molar heme ellipticity, MHE) (Fig. 7*D*). The monoheme m4D2 exhibited only a positive Cotton effect in this region indicating the heme was held in an asymmetric environment and that no heme–heme exciton coupling was present (52). In contrast, both 4D2 and e4D2 exhibited split Cotton effects indicative of strong exciton coupling between hemes in confined environments (53), with a higher intensity of the signal in e4D2. Interestingly, the visible CD spectra of 4D2 and e4D2 strongly resemble those of cytochrome *b* from the cytochrome *bc*_1_ complex (53, 54), suggesting that the relative orientations of hemes are similar to those of cytochrome *b* (26). Comparing 4D2 with e4D2, the increased signal intensity indicates an increased dipolar coupling strength and thus a greater dissociation of the excited state over the larger heme chain. However, further computational modelling will be necessary to reveal the precise heme orbital effects of increasing the number of cofactors within such tightly packed systems and how this may impact their redox properties. Our expandable platform of heme proteins is primed to address such questions, providing a versatile testbed for varying heme–heme distances and local heme electrostatic environments.

### Precise, Predictable Redox Engineering.

Since the ability to precisely specify and modulate the midpoint potentials of redox sites and cofactors within proteins would be an exceptionally valuable tool for protein engineers, we decided to use m4D2 as a testing ground for integrating predictive redox calculations into our design process. To this end, we used a well-established continuum electrostatics method, combining Poisson–Boltzmann calculations and Monte-Carlo simulations (PB–MC) (25, 55) to predict shifts in the heme redox potentials between static computational models of two m4D2 mutants (T19D & T19D/T77D) relative to m4D2. As described above, the m4D2 and related models were obtained through computational redesign of 4D2. The mutants were selected due to their potential electrostatic influence around the heme-binding site, a critical factor in heme redox potential.

Previous work with natural peroxidases has established that aspartate residues local to the proximal heme-ligating histidine play key roles in modulating the imidazolate character of the histidine side chain (56, 57), priming the heme for catalysis by maintenance of the correct histidine tautomeric state. This increase in local electronegativity results in a pronounced negative shift of the heme midpoint potential. Therefore, for m4D2, we initially selected the two threonine residues involved in the keystone hydrogen bonding interactions with the coordinating histidines (24) for mutation to aspartate, and constructed both single- and double-mutant variants (T19D & T19D/T77D). After obtaining Rosetta-relaxed structures of m4D2 and these mutants, a single conformation of each variant was used for the PB–MC calculations. While we were unable to obtain high-resolution structures of m4D2 or its mutants, our aforementioned structure of 4D2 with the equivalent T19D mutation demonstrated that there is almost no structural perturbation following the threonine to aspartate substitution (Fig. 7*E*); the isosteric aspartate side chain occupies the same volume as the original threonine side chain, and H-bonds to the heme-coordinating histidine. The PB–MC calculations predicted shifts in E_m_ of −23 mV for the single aspartate mutant and −58 mV for the double aspartate mutant (Fig. 7*F*) relative to the original m4D2 protein. When we expressed and experimentally characterized these mutants, we observed shifts of −27 mV and −56 mV, respectively (Fig. 7*G*), in remarkable agreement with the values predicted by the PB–MC calculations.

We then created and experimentally characterized (Fig. 7*H* and *SI Appendix*, Figs. S14 and S15) four additional mutants centered around the heme, targeting hydrophobic interactions with the heme (M23N & M23Q), and electrostatic interactions between the heme propionates and positively charged arginines (R34Q & R92Q) (*SI Appendix*, Fig. S16). The work on the M23N, R34Q, and R92Q mutants was performed in parallel with another study designed to predict absolute redox potentials from MD simulations, in which PB–MC-derived redox potential shifts are also reported (58); however, in the work reported here, we included an additional energy minimization step in Rosetta prior to performing the calculations. There was a good correlation between the PB–MC predictions and the experimentally determined redox potential shifts [correlation coefficient (*ρ*) of 0.935, Fig. 7*I*]. Higher correlation coefficients might be obtained by including protein dynamics into the calculations, achieved through calculation of the average redox potentials of variants through MD simulations (58). Nevertheless, these results demonstrate the utility of building such property prediction tools into a design pipeline, enabling modulation and fine-tuning of redox potential for de novo proteins.

## Conclusions

With this work, we have attempted to address the deficiencies in the precision design of de novo redox proteins by creating a framework of expressible, well folded single and multiheme proteins. A key feature of our design process and our platform is the ability to predictably design and obtain cofactor-containing proteins with well-defined structures; without this being fully realized, it has not been possible to gain fine control of protein and cofactor properties, and this has ultimately hindered the exploitation of these highly desirable cofactors, proteins and enzymes in biotechnological and bionanotechnological applications. Another is modularity; our approach will enable individual parts to be designed and then combined in desirable arrangements for specific functions. Though currently sparse in number, there are other examples of such modular design approaches, where tetrapyrrole binding sites have been combined with binuclear metal binding sites to explore allosteric regulation of catalytic function (13) and photoactivated charge separation (12, 59). Both studies, like ours, combined de novo designed cofactor binding domains into extended tetrahelical bundles. To fully unlock the diverse functional repertoire of the natural oxidoreductases, it will be necessary to integrate MD simulations and continuum electrostatics calculations, and other prediction tools, into the design process to define and modulate biophysical properties such as redox potentials (60), as we have described here for the 4D2 family of de novo proteins. Future work will thus focus on further modulating redox potential, both in monoheme proteins and within modular multiheme chains, enabling, for example, the imposition of directionality of electron transfer in these proteins. Such endeavors will open the door to new biological and nanotechnological applications for these and other de novo designed proteins, inspired by the natural electron-transferring circuitry of life, but tailor made for incorporation into bioelectronic devices and the bioenergetic pathways of living organisms or synthetic protocells.

## Materials and Methods

Additional experimental methods are provided in *SI Appendix*.

### X-Ray Crystallography.

Crystals suitable for data collection were obtained by the sitting drop vapor diffusion method at 22 ℃ using the Morpheus protein crystallization screen (Molecular Dimensions Ltd). Protein samples were concentrated to between 10 and 20 mg/mL and mixed in a 1:1 ratio with precipitant in 1 μL sitting drops. X-ray diffraction data were collected at beamline I03 (Diamond Light Source). A fluorescence edge scan was measured to determine required wavelengths for phase solving by MAD, collecting diffraction data at peak, inflection, and high remote wavelengths (1.738 Å, 1.741 Å, and 1.698 Å). Diffraction data were integrated and scaled with XDS and XSCALE (61) using xia2 (62) prior to phasing and model refinement in the CCP4i2 (63) software suite. Experimental phasing by MAD was performed using SHELX (64), followed by iterative rounds of model building and refinement using Refmac5 (65) and Coot (66). 4D2 crystallized in the H3 space group with unit cell dimensions a = 81.25 Å, b = 81.25 Å, and c = 59.06 Å. The T19D variant crystallized in the same space group (H3) with unit cell dimensions a = 81.09 Å, b = 81.09 Å, and c = 58.89 Å. The refined coordinates have been deposited to the PDB [ID: 7AH0 (4D2), 8CCR (4D2 T19D)], and data collection and refinement statistics are available in *SI Appendix*, Table S1.

### NMR Spectroscopy.

Isotopically labeled protein for NMR was expressed in minimal media following a modified version of the protocol outlined in Rupasinghe et al. (67). The unlabeled minimal media contained 18 mM NH_4_Cl, 4 g/L glucose, 34 mM Na_2_HPO_4_, 22 mM KH_2_PO_4_, 86 mM NaCl, 2 mM MgCl_2_, 50 μM FeCl_3_, 20 μM CaCl_2_, 10 μM MnCl_2_,10 μM ZnSO_4_, 2 μM CuCl_2_, 2 μM CoCl_2_, 2 μM NiCl_2_, and 2 μM H_3_BO_3_. Labeled media contained the same constituents except for the substitution of ^15^NH_4_Cl and ^13^C-Glucose (Cambridge Isotopes) for their unlabeled counterparts. The cells were grown in unlabeled minimal media to an OD_600_ of 0.6. The cells were then harvested by centrifugation at 4,000 xg and resuspended in labeled media. The cultures were incubated at 28 ℃ for one hour before the addition of IPTG (final concentration of 0.5 mM). Expression was carried out for 18 h at 28 ℃. Labeled samples were purified as described above.

Purified protein was resuspended in ~3 mL of borate buffer (100 mM boric acid, 250 mM NaCl pH 9.0) to a final concentration of ~50 μM. The complex was formed by the addition of 0.1 molar equivalents every ten minutes of a stock solution of a symmetric variant of heme, iron 2,4-dimethyl-deuteroporphyrin IX (Frontier Scientific). The stock solution was prepared at 5 mg/mL in DMSO. The additions were repeated until a total of 1.5 molar equivalents was added to ensure complete binding. The sample was then spin concentrated to a final volume of ~500 μL and buffer exchanged into a suitable buffer for NMR (20 mM KH_2_PO_4_ 50 mM KCl pH 6.4 10% D_2_O) using a PD-10 column (GE Healthcare). The concentration of the final NMR sample was typically 0.4 to 1 mM.

NMR spectra were acquired at 25 °C or 35 °C with a 700 MHz Bruker Avance III HD instrument (BrisSynBio NMR facility) equipped with a 1.7 mm triple-resonance microcryoprobe, or a Bruker Avance III HD 800 MHz NMR Spectrometer (New York Structural Biology Center, NYSBC) with a 5-mm cryoprobe. Spectra were analyzed using CCPNmr Analysis7 Version 2.4. The backbone resonances were assigned using HNCA/HNCOCA/HNCACB/ HNCOCACB and HNCO/HNCACO spectra. Side-chain resonances were assigned using CCONH, ^15^N-edited TOCSY, ^15^N-edited NOESY, HCCH-TOCSY, and ^13^C-edited NOESY spectra. All the NMR data were processed using NMRPipe (68) and the spectra were visualized and assigned using CCPNmr Analysis version 2.4.2 (69). The chemical shift assignments for >90% of the backbone residues were obtained using a combination of autoassign with I-PINE (70) and manual analysis. Unassigned residues were found in the N terminus and the flexible loop regions.

### Negative-Stain Sample Preparation and Electron Microscopy.

Five ml of 0.02 mg/mL of e4D2 protein sample was applied onto a freshly glow-discharged (1 min at 15 mA) CF300-Cu grid (Electron Microscopy Sciences), incubated for 1 min, and manually blotted. Five mL of 3% uranyl acetate was applied onto the same grid and incubated for 1 min before the solution was blotted off. Images were acquired at a nominal magnification at 80,000× magnification corresponding to a pixel size of 1.27 Å/pix. A total of 25,025 particles using RELION 3.1 (47) from 200 images were picked, and reference free two-dimensional classification was performed leading to 13,396 particles included in final 2D class averages (*SI Appendix*, Fig. S11).

### Cryo-EM Sample Preparation and Data Collection.

Three μl of 2.0 mg/mL of e4D2 sample was loaded onto a glow-discharged Quantifoil R1.2/1.3 holey carbon grid (Agar Scientific). The sample was incubated for 30 s at 90% relative humidity and 16 °C inside a Leica EM ACE 600 (Leica EM GP2 plunge freezer), blotted for 1.2 s and plunge frozen. Cryo-EM data were collected at 200 kV with a FEI Talos Arctica microscope equipped with a Gatan K2 Summit direct electron detector and a Gatan Quantum GIF energy filter operated in zero-loss mode with a slit width of 20 eV using the automated acquisition software (EPU). A total of 5,606 dose-fractionated movies were recorded in counted superresolution mode at a nominal magnification of 130,000× corresponding to a physical pixel size of 1.05 Å and a virtual pixel size of 0.525 Å with a defocus range of −1.2 to −2.4 μm (*SI Appendix*, Fig. S12 and Table S2). Each movie contained 46 frames (0.25 s per frame) with an accumulated total dose of 62 e−/Å^2^.

### Cryo-EM Data Processing.

Image processing was performed using the RELION 3.1 software package (47). The dose-fractionated movies were gain normalized, aligned, and dose-weighted using MotionCor2 (71) and contrast transfer function (CTF) information determined and corrected using Gctf find4.1 (72). A total of 2,888 micrographs with CTF rings extending beyond 6 Å were selected for further processing. 1,392,111 particles were autopicked using RELION autopicking software. Several rounds of reference-free 2D classification (*SI Appendix*, Fig. S12) were performed, followed by initial 3D-autorefinement with well-defined particles. This initial refinement applied D2 symmetry based on the pseudosymmetry of the helical bundle and heme cofactors, generating an initial 3D reference map which was utilized for subsequent 3D refinements. Further rounds of 3D-classification/refinement were carried out on 85,654 particles after applying D2 symmetry. The D2 symmetrized map was then used as reference for further 3D classification without applying any symmetry. This was followed by reference-free 2D classification yielding 13,303 particles for final 3D refinement (C1, no symmetry), and global resolution and B-factor (−262 Å^2^) of the maps were estimated by applying a soft mask around the protein density. The final map reached an overall resolution of 8.4 Å using the gold-standard FSC criterion 0.143 (*SI Appendix*, Fig. S13).

### Computational Design of m4D2 and e4D2.

The computational design of the monoheme m4D2 structure was performed using Rosetta (versions 3.8 to 3.11) to repack the protein core, executed via Rosetta scripts (39). Partial charges for ferrous heme in the CHARMM parameters were used to construct a Rosetta heme parameter file, and the Fe-N_ε_ distance of the heme–histidine coordination was constrained during design (harmonic, distance = 2.1 Å, SD = 0.01). A model of e4D2 was built by extracting coiled-coil parameters of opposing helices in the 4D2 crystal structure using ISAMBARD (45), and new helices generated with the extended sequence using these parameters. These helices were oriented by alignment with 4D2 to position the four heme cofactors, then missing loops were added and the backbone structure minimized in Rosetta.

### MD.

All MD simulations were performed in AMBER16 (73). The Amber ff14SB forcefield was used to describe the protein, while parameters derived for the bis-histidine ligated *b*-type heme in *b*-type heme-containing cytochrome *c* oxidase (74, 75) were used for the cofactor. The models designed using Rosetta (see previous section for more details) were used as the starting points for the MD simulations. Structures were prepared in tleap, solvated in a TIP3P cubic box with a minimum 10-Å distance between the protein and edge of the box. All simulations were run using the pmemd.cuda application on University of Bristol HPC clusters (Bluecrystal phase 3, 4, BlueGem). Trajectories were analyzed using cpptraj, with triplicate 100-ns-long trajectories of apo-m4D2 used to assess design stability.

As stated in the *Data, Materials, and Software Availability* section, all computational design (Rosetta, ISAMBARD) and MD (AMBER) input files and parameters are available in the following GitHub repository: https://github.com/georgehutch/4D2computational, and all computational data and the associated analyses are available from the University of Bristol data repository (https://doi.org/10.5523/bris.3crx74ryps8h42aol8pbjycetp).

### Continuum Electrostatics Calculations (PB–MC).

The shift in redox potential of the heme group between m4D2 and corresponding mutants was determined using methodologies for studying the thermodynamics of proton and electron binding described previously (25, 76). These methods consist of simulating the joint-binding equilibrium of proton and electrons using a combination of Poisson–Boltzmann (PB) calculations and Metropolis Monte Carlo (MC) simulations. The PB calculations compute the individual and pairwise terms needed to obtain the free energies of protonation/reduction changes. Such energies are then used in the MC simulation. The E_m_ shift of the heme group relative to m4D2 is then determined from the corresponding redox curve.

The PB calculations were performed with the software MEAD (77–79), whereas the MC simulations were done with the software PETIT (80). The atomic charges for all the atoms in the protein (except the heme group) and radii were taken from the GROMOS 54A7 force field (81) using a previously described procedure (82). All of the simulations used a temperature of 298 K and a molecular surface defined with a solvent probe radius of 1.4 Å. The dielectric constants used for the solvent and protein were 80 and 20 (82), respectively. Each MC simulation comprises 10^5^ MC steps, and the acceptance/rejection of each step followed a Metropolis criterion (83) using the previously determined PB free energies.

The partial charges for the oxidized and reduced states of the heme B group (including the axial histidine sidechains coordinating the iron atom up to the C_β_ atom) were determined using quantum chemical methods (*SI Appendix*, Table S3). The propionate groups were excluded from the quantum calculations, and, therefore, replaced by methyl groups (*SI Appendix*, Fig. S17), similarly to Oliveira et al. (75). All quantum calculations were performed using GAUSSIAN09. Energy optimization of all the protons was performed with fixed hetero-atoms using B3LYP basis set, and the 6-31G(d) and 6-31G(3df) basis sets for heteroatoms and iron atoms, respectively. The resulting optimized structures were used for single-point calculations using B3LYP and cc-pVTZ basis sets, with the inclusion of solvent effects with a polarizable continuum model and a dielectric constant of 4. The resulting electrostatic potentials were then fitted to the models using RESP (84) to all the atoms except iron, nonpolar, and nonaromatic hydrogens (in a united-atom approach). To avoid artifacts associated with RESP fitting on buried atoms, the charges of the iron atoms were kept at their Mulliken partial charge value as in Oliveira (75).

## Supplementary Material

Appendix 01 (PDF)

## Data Availability

All computational protein design (Rosetta, ISAMBARD) and MD (AMBER) input files and parameters are available in a GitHub repository (https://github.com/georgehutch/4D2computational) (85), and all computational data, the associated analyses, and the cryo-EM maps are contained within the University of Bristol data repository (https://doi.org/10.5523/bris.3crx74ryps8h42aol8pbjycetp) (86). X-ray crystallographic coordinates and associated data files were deposited in the Protein Data Bank (PDB) with accession codes 7AH0 (4D2) (87) and 8CCR (4D2 T19D) (88). The cryo-EM map was deposited in the Electron Microscopy Data Bank with accession code EMD-16847 (e4D2) (89). All other data are included in the article and/or *SI Appendix*.
