# Supplementary material for: An expandable, modular de novo protein platform for precision redox engineering
Source: Proc Natl Acad Sci U S A. 2023 Jul 24;120(31):e2306046120. doi: 10.1073/pnas.2306046120 (PMC10400981; doi:10.1073/pnas.2306046120)
Supplement: Supplementary file 1 — Appendix 01 (PDF) [file pnas.2306046120.sapp.pdf]

Supplementary Information for:

An expandable, modular *de novo* protein platform for precision redox engineering

*George H. Hutchins[1], *Claire E. M. Noble[1,2], H. Adrian Bunzel[1], Christopher Williams[3], Paulina Dubiel[1], Sathish Yadav Kadapalakere[1], Paul M. Molinaro[4,5], Robert Barringer[1], Hector Blackburn[1], Benjamin J. Hardy[1], Alice E. Parnell[1,2], Charles Landau[1], Paul R. Race[1,2], Thomas A. A. Oliver[3], Ronald L. Koder[4,5], Matthew P. Crump[3], Christiane Schaffitzel[1], A. Sofia F. Oliveira[1,3], Adrian J. Mulholland[1,2,3], J. L. Ross Anderson[1,2].

[1]School of Biochemistry, University of Bristol, University Walk, Bristol, BS8 1TD, UK.
[2]BrisSynBio Synthetic Biology Research Centre, Life Sciences Building, University of Bristol, Tyndall Avenue, Bristol BS8 1TQ, UK.
[3]School of Chemistry, University of Bristol, Bristol, BS8 1TS, UK.
[4]Department of Physics, The City College of New York, New York NY10031
[5]Graduate Programs of Physics, Biology, Chemistry and Biochemistry, The Graduate Center of CUNY, New York, New York 10016, United States
* *these authors contributed equally to this work*

email: ross.anderson@bristol.ac.uk

Contents.

Supplementary Figure 1: DNA and Amino Acid sequences of 4D2, m4D2 and e4D2.
Supplementary Figure 2: 4D2 expression and *in vivo* heme loading.
Supplementary Figure 3: Mass spectrometry of m4D2, 4D2 and e4D2.
Supplementary Figure 4: 2D NMR spectroscopy of 4D2 with heme B and Fe(III) DMDPIX.
Supplementary Figure 5: Crystal structures of 4D2 and 4D2 T19D.
Supplementary Figure 6: Crystal structures reveal uncertainty in heme B orientations within the 2 binding sites.
Supplementary Figure 7: Analysis of MD simulations of apo-m4D2, holo-m4D2 and holo-e4D2 designs.
Supplementary Figure 8: ^{1}H-^{15}N HSQC NMR spectrum of m4D2 loaded with heme B.
Supplementary Figure 9: Binding isotherm of apo-m4D2 versus Fe(III) DMDPIX.
Supplementary Figure 10: Size exclusion chromatography of m4D2, 4D2 and e4D2.
Supplementary Figure 11: Negative stain electron microscopy analysis of e4D2.
Supplementary Figure 12: Flowchart of e4D2 cryo EM processing in Relion 3.0.
Supplementary Figure 13: Fourier shell correlation statistics for the e4D2 cryo-EM structure analysis.
Supplementary Figure 14: Far-UV CD spectra of heme-loaded m4D2 variants with varying temperature.
Supplementary Figure 15: Redox potentiometry of m4D2 M23Q.
Supplementary Figure 16: Positions for redox altering m4D2 mutations mapped onto the 4D2 crystal structure.
Supplementary Figure 17: Schematic representation of the bis-histidine heme B redox center.
Supplementary Table 1: X-ray data collection and structural refinement statistics.
Supplementary Table 2: Cryo-EM data collection and structural refinement statistics.
Supplementary Table 3: Calculated atomic partial charges for bis-histidine ligated heme B.
Supplementary Methods
References

All constructs have an upstream His tag, V5 epitope and a TEV cleavage site:
Amino acid (AA) sequence: MHHHHHHGKPIPNPLLGLDSTENLYFQ
DNA sequence: ATGCATCATCACCATCACCATGGTAAGCCTATCCCTAACCCTCTCCTCG
GTCTCGATTCTACGGAAAACCTGTATTTTCAG

>4D2
Vector: pET151
AA sequence (112 aa):
GSPELREKHRALAEQVYATGQEMLKNTSNSPELREKHRALAEQVYATGQEMLKNGSVSPSPELREKHRALAEQV
YATGQEMLKNTSNSPELREKHRALAEQVYATGQEMLKN
DNA sequence:
GGATCGCCAGAACTGCGCGAGAAACACCGTGCGTTAGCCGAACAAGTGTACGCCACAGGCCAAGAAATGCT
GAAGAACACGAGCAATTCGCCGGAACTTCGCGAGAAACATCGTGCTCTGGCAGAACAGGTGTATGCGACTG
GCCAGGAAATGCTGAAAAACGGGTCTGTAAGTCCGTCACCTGAACTGCGGGAGAAACACCGCGCTTTGGCC
GAACAGGTTTACGCAACCGGTCAGGAGATGCTCAAGAACACCTCCAATAGCCCGGAACTGCGTGAGAAACA
TCGCGCATTAGCGGAACAAGTCTATGCGACCGGTCAGGAAATGTTGAAAAAT

>m4D2
Vector: pET151
AA sequence (112 aa):
GSPELREKLRALIEQVYATGQEMLKNTSNSPELREKHRALAEQVYATWQELLKNGSVSPSPELREKFRALLEQVY
ATGQEMLKNTSNSPELREKHRALAEQVIATWQELLKN
DNA sequence:
GGAAGTCCGGAACTTCGTGAAAAACTGCGTGCACTGATTGAACAGGTTTATGCAACCGGTCAGGAAATGCTG
AAAAATACGAGCAATAGCCCTGAGCTGCGCGAGAAACATCGCGCCCTGGCAGAGCAAGTCTACGCCACGTG
GCAAGAACTGTTAAAGAACGGTAGCGTTTCTCCGTCACCAGAATTACGCGAAAAATTTCGGGCGCTTCTGGA
ACAAGTGTATGCCACAGGCCAAGAGATGCTTAAAAACACCTCGAACTCTCCTGAGCTGCGGGAAAAGCACCG
TGCATTAGCCGAGCAGGTTATTGCGACTTGGCAGGAATTACTGAAGAATTGA

>e4D2
Vector: pET151
AA sequence (196 aa):
GSPELREKHRALAEQVYATGQEMLELREKHRALAEQVYATGQEMLKNTSNSPELREKHRALAEQVYATGQEMLE
LREKHRALAEQVYATGQEMLKNGSVSPSPELREKHRALAEQVYATGQEMLELREKHRALAEQVYATGQEMLKNT
SNSPELREKHRALAEQVYATGQEMLELREKHRALAEQVYATGQEMLKN
DNA sequence:
GGATCTCCCGAATTACGCGAGAAACACCGCGCTCTTGCTGAGCAGGTATATGCGACTGGCCAGGAAATGCT
TGAGCTGCGGGAGAAACACCGTGCGTTAGCCGAACAAGTGTACGCGACAGGGCAGGAAATGCTGAAGAATA
CCTCCAATTCGCCTGAGTTGCGTGAAAAGCACCGCGCGCTTGCGGAACAGGTGTATGCGACTGGTCAGGAG
ATGTTGGAACTGCGCGAGAAACATCGTGCCCTCGCAGAACAGGTTTACGCAACTGGTCAGGAAATGTTGAAG
AATGGGAGCGTTAGCCCTAGTCCGGAACTGCGTGAGAAACACCGGGCTTTGGCAGAACAGGTATATGCCAC
TGGACAGGAGATGCTGGAACTCCGCGAAAAGCATCGCGCACTGGCTGAACAAGTTTACGCTACAGGCCAAG
AGATGCTTAAGAATACCTCGAATTCTCCGGAACTCCGTGAGAAACATCGCGCCCTGGCGGAACAGGTCTATG
CTACGGGTCAAGAAATGCTGGAGTTACGGGAAAAGCACCGTGCCCTGGCTGAGCAGGTCTATGCAACGGGA
CAAGAGATGCTGAAGAACTAA

Supplementary Figure 1: DNA and Amino Acid sequences of 4D2, m4D2 and e4D2.

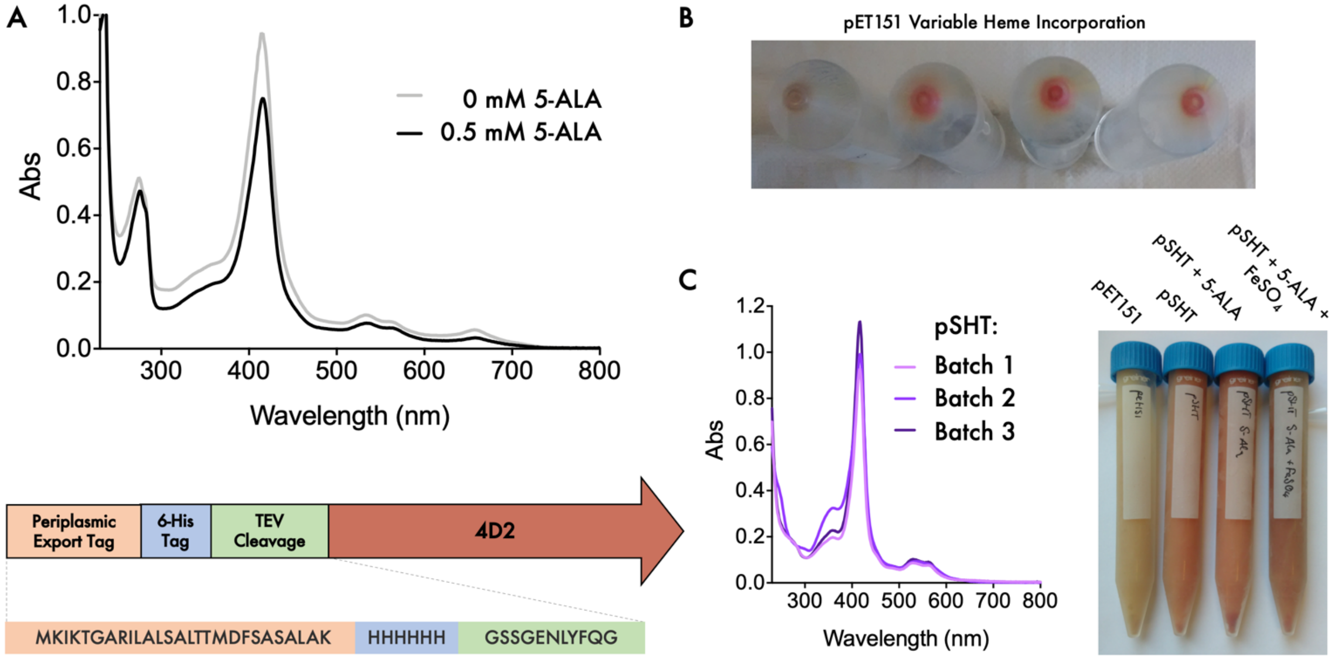

Supplementary Figure 2: 4D2 expression and *in vivo* heme loading. **A.** The addition of δ-aminolevulinic acid can benefit heme loading, though the effect is inconsistent; cells often manage to achieve high levels of heme B loading in its absence. **B.** Centrifuged cell pellets from identically treated 4D2-expressing cultures in the absence of δ-aminolevulinic acid highlight the stochastic, unpredictable heme B-loading in cells. **C.** Expression of 4D2 in the *E. coli* periplasm from the pSHT vector alleviated the unpredictable heme loading, and removed the requirement for supplementation.

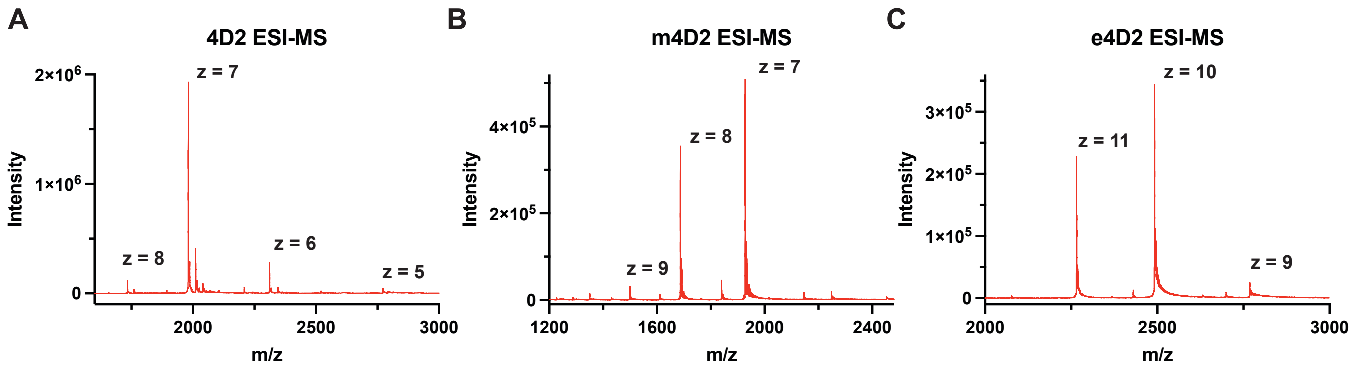

D

Protein	Expected mass (Da)	Actual mass (Da)	Mass difference
m4D2 (+ 1 heme B)	13490.8	13488.9	-1.9
4D2 (+ 2 heme B)	13864.9	13865.1	0.2
e4D2 (+ 4 heme B)	24914.0	24916.0	2.0

Supplementary Figure 3: Mass spectrometry of m4D2, 4D2 and e4D2. **A, B, C.** ESI-MS spectra of the three proteins, with labels indicating major ionization states of fully heme-loaded versions. **D.** Holoprotein masses calculated from analysis of the ESI-MS spectra.

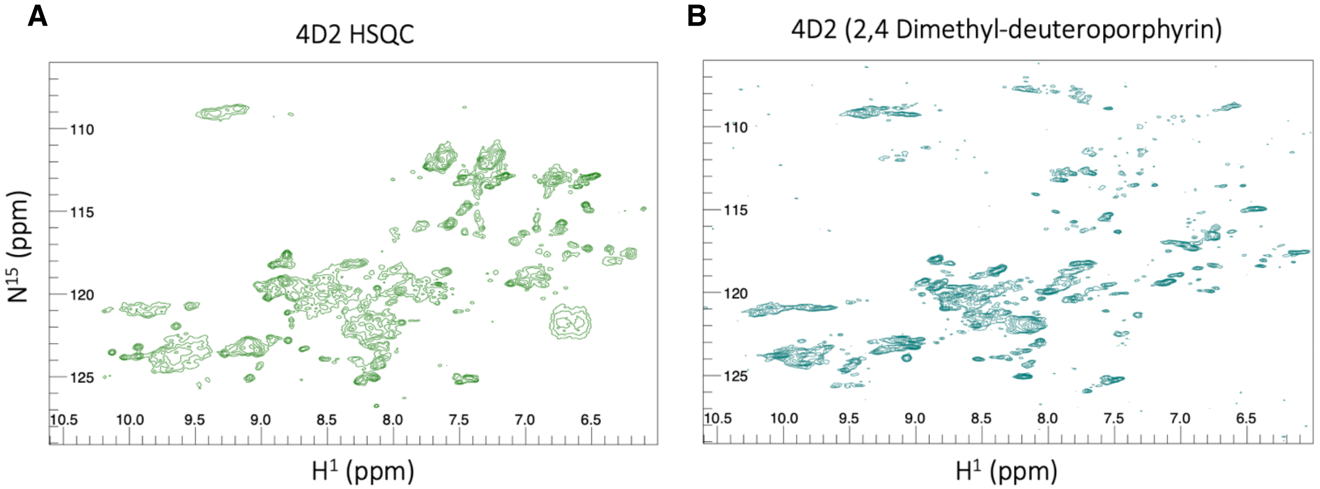

Supplementary Figure 4: 2D NMR spectroscopy of 4D2 with heme B and Fe(III) DMDPIX. 4D2 ^{1}H-^{15}N HSQC spectrum (**A**) demonstrates moderate peak dispersion, insufficient for sequence assignment or further structural analysis. Reconstitution with a symmetric porphyrin (**B**) results in a significant shift, offering further evidence for multiple heme binding states, but does not improve the spectrum quality to the same extent as observed for the monoheme variant m4D2.

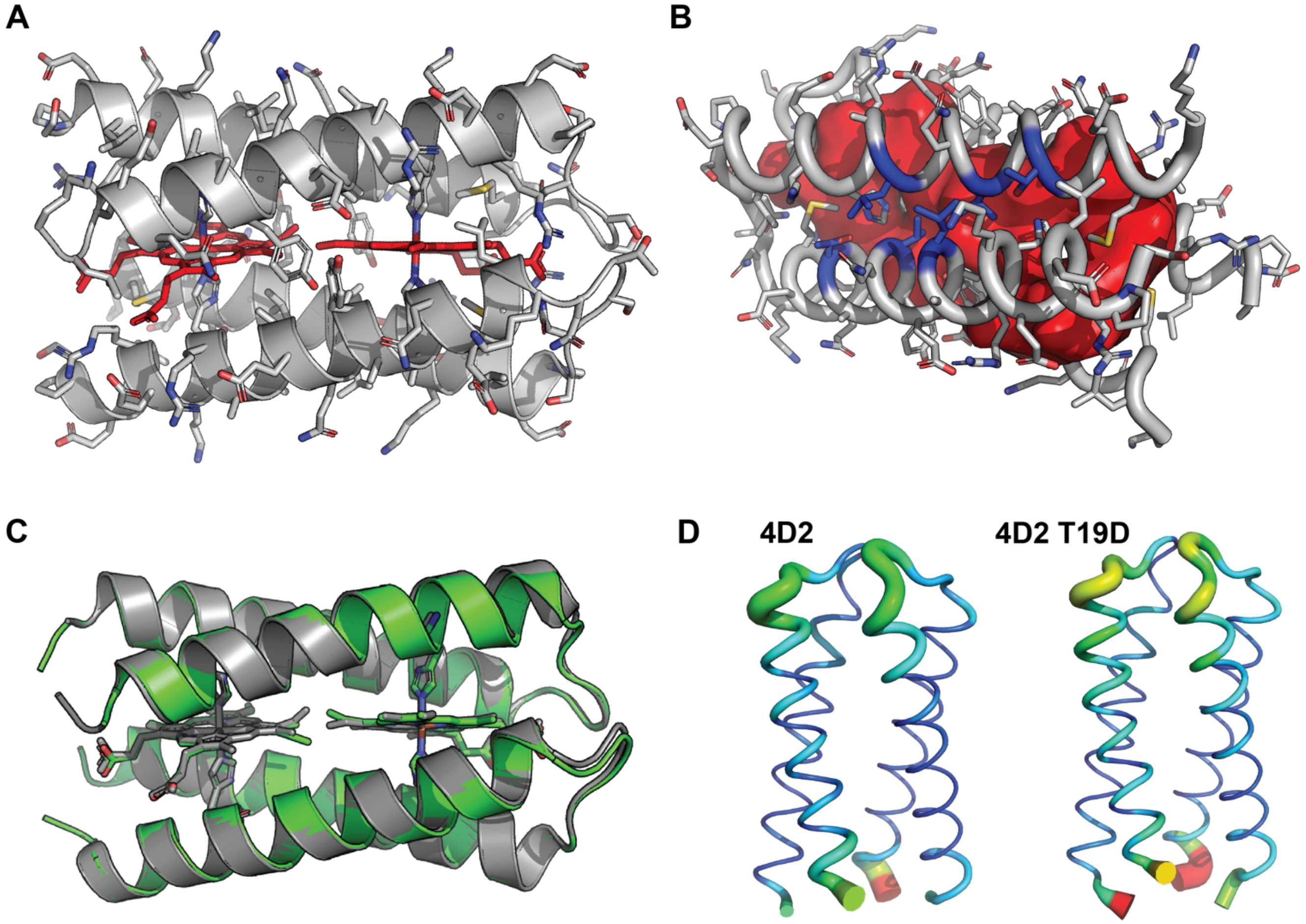

Supplementary Figure 5: Crystal structures of 4D2 and 4D2 T19D. **A.** The 1.91 Å structure of 4D2, highlighting amino acid side chains and heme positioning. **B.** Representation of the inner, heme-binding cavity of 4D2, that dominates the protein core and almost separates the four-helix bundle into two dimeric coiled coils. **C.** Overlay of 4D2 (green) and 4D2 T19D (grey) structures shows minimal structural rearrangement on mutation. **D.** Crystallographic B-factors mapped onto the structures of 4D2 (left) and 4D2 T19D (right).

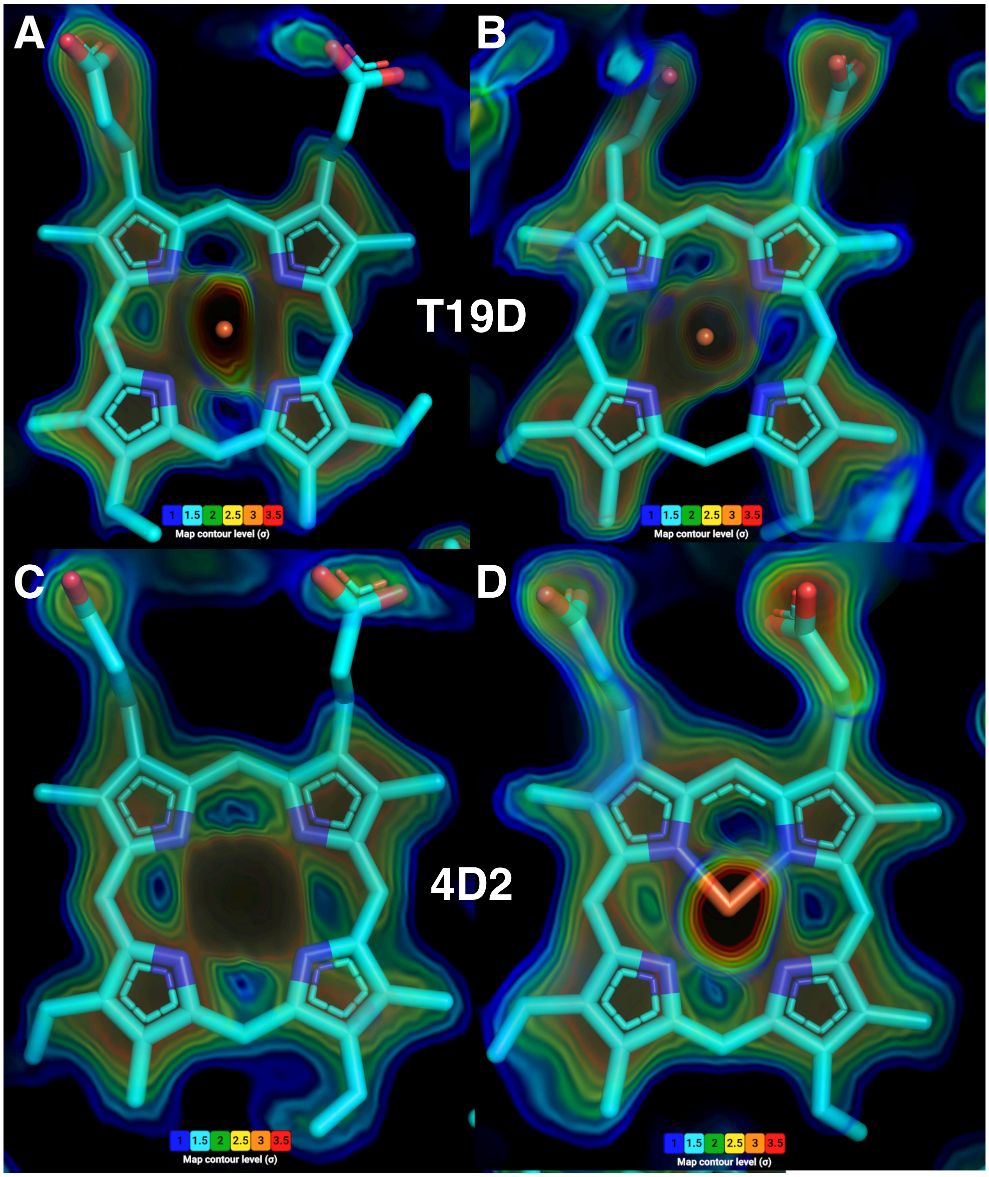

Supplementary Figure 6: Crystal structures reveal uncertainty in heme B orientations within the two binding sites. $2F_O$-$2F_C$ OMIT maps of the heme B molecules in the 4D2 T19D (**A**, **B**) and 4D2 (**C**, **D**) crystal structures, coordinated by the H9/H67 (**A**, **C**) and H37/H95 (**B**, **D**) pairs. Contour σ values are indicated by the scale at the bottom left of the figures.

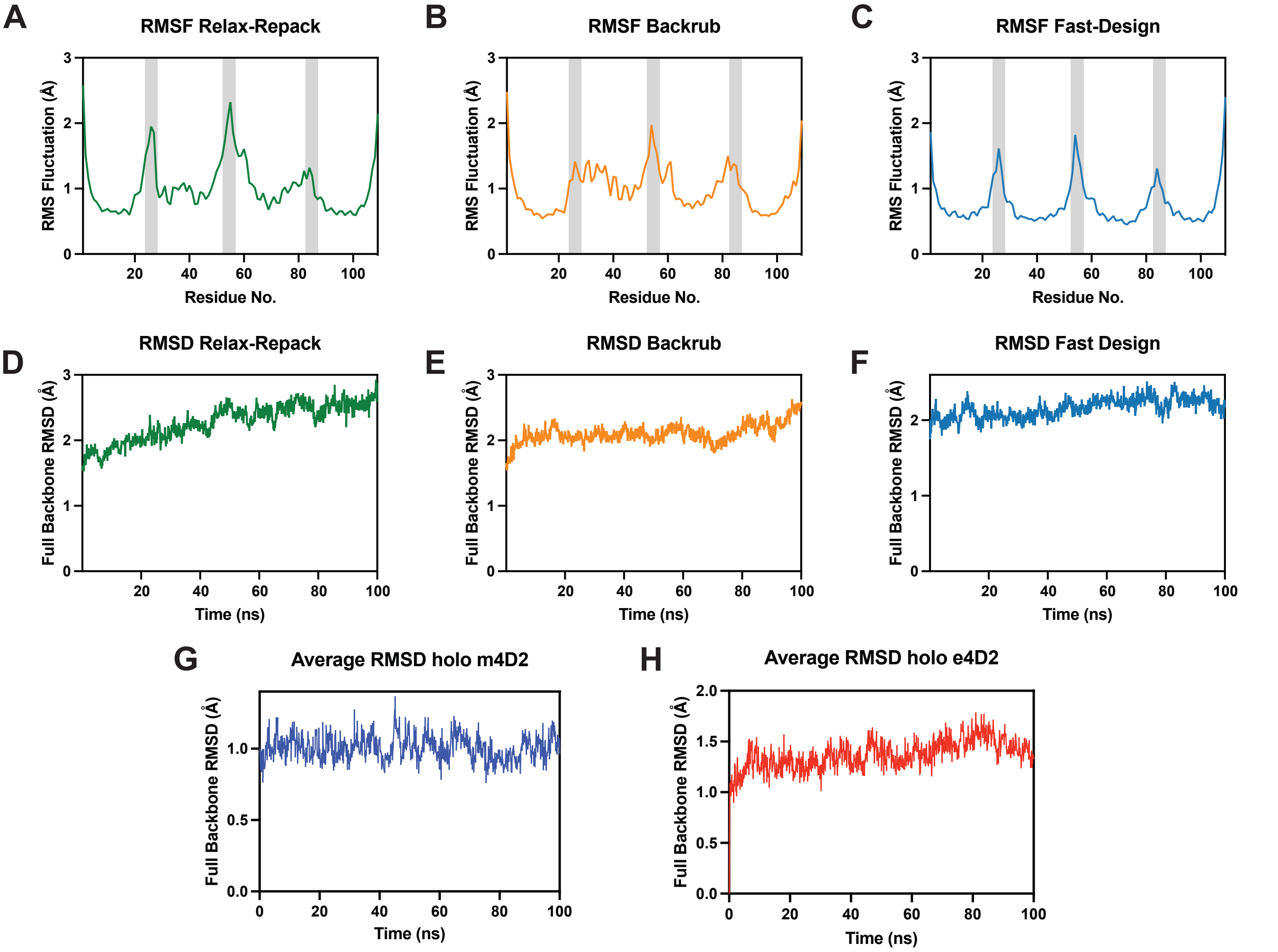

Supplementary Figure 7: Analysis of Molecular Dynamics simulations of apo-m4D2, holo-m4D2 and holo-e4D2 designs. **A, B, C.** Average root mean square fluctuations (RMSF) calculated per residue for each apo-m4D2 design highlighted the flexible regions of the protein, such as the loops and empty heme binding site. Grey rectangles indicate the loop regions of the proteins. **D, E, F.** Average root mean square deviations (RMSD) for all backbone atoms demonstrated that the Backrub and Fast-Design structure deviated least from the designed apo-m4D2 model, and remained relatively rigid throughout the simulation. **G.** Average root mean square deviations (RMSD) for all backbone atoms of holo-m4D2 designed using the Backrub protocol. **H.** Average root mean square deviations (RMSD) for all backbone atoms of the holo-e4D2 design.

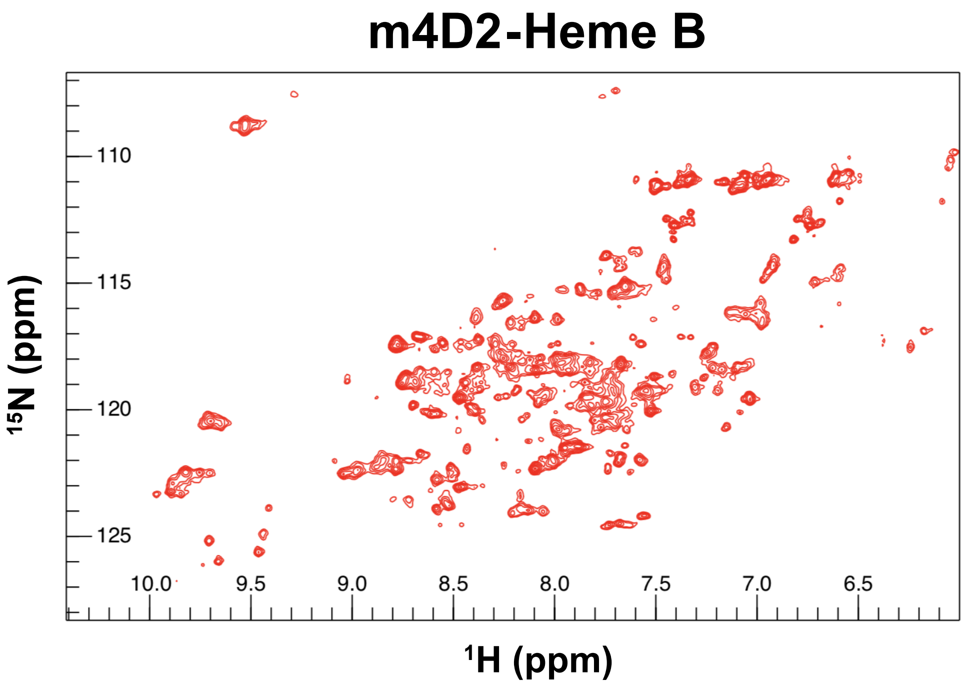

Supplementary Figure 8: ^{15}N-^{1}H HSQC NMR spectrum of m4D2 loaded with heme B, demonstrating improved signal dispersion compared with that of 4D2 in Supplementary Fig. 4.

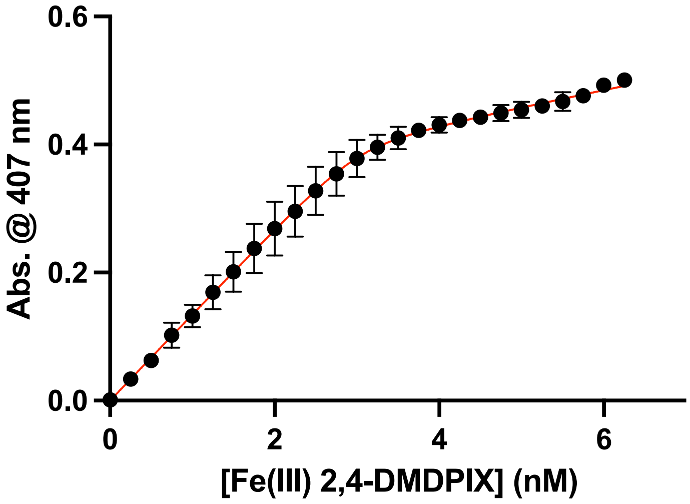

Supplementary Figure 9: Binding isotherm of apo-m4D2 (3 µM in 20 mM CHES, 100 mM KCl, pH 8.6) versus Fe(III) DMDPIX in DMSO. Data was recorded in triplicate, with *error bars* representing the standard deviation. The dissociation constant (K_D) of 25 nM indicates a slight decrease in affinity versus heme B, but still in the low nanomolar range.

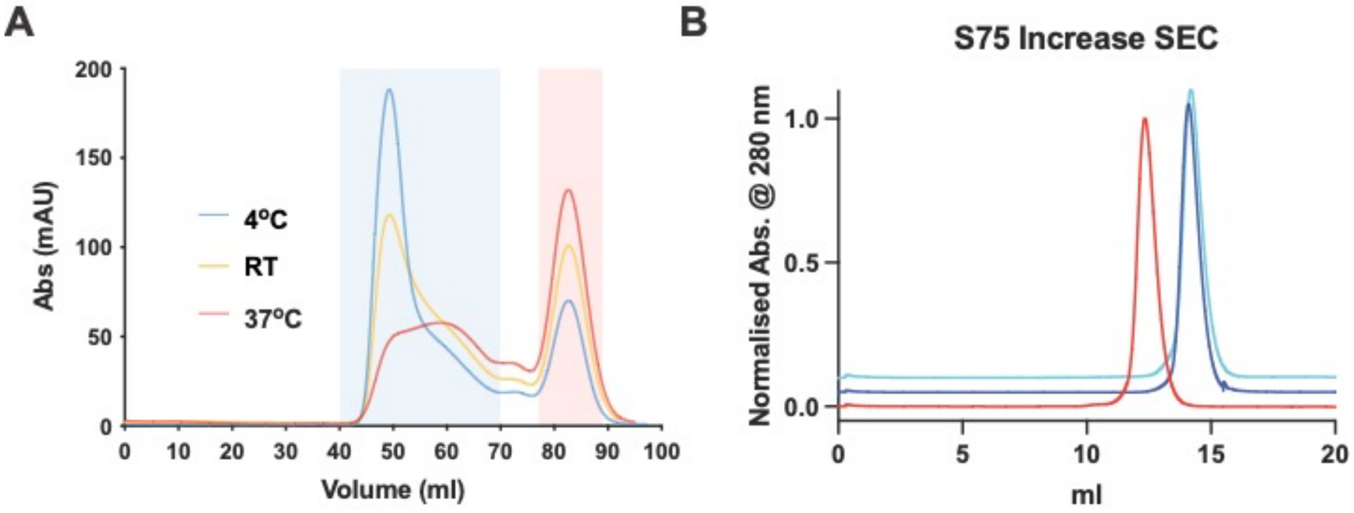

Supplementary Figure 10: Size exclusion chromatography of m4D2, 4D2 and e4D2. **A.** The elution profile of e4D2 is dependent on the temperature at which heme B is loaded, with higher temperatures resulting in a larger quantity of the monomeric e4D2, highlighted in the pale red band at approximately 82 mL elution volume. Data were acquired using a HiLoad 16/600 Superdex S75 pg column (Cytiva), equilibrated with 20 mM CHES, 100 mM KCl, pH 8.6. **B.** Analytical size exclusion chromatography of heme loaded and purified m4D2 (blue), 4D2 (cyan) and e4D2 (red), demonstrating monodisperse monomeric species for each, and the absence of higher order oligomers or aggregates in the samples used for biophysical analysis. Chromatograms are offset from each other for clarity. Data were acquired using a Superdex 75 Increase 10/300 GL column (Cytiva) equilibrated with 20 mM CHES, 100 mM KCl, pH 8.6.

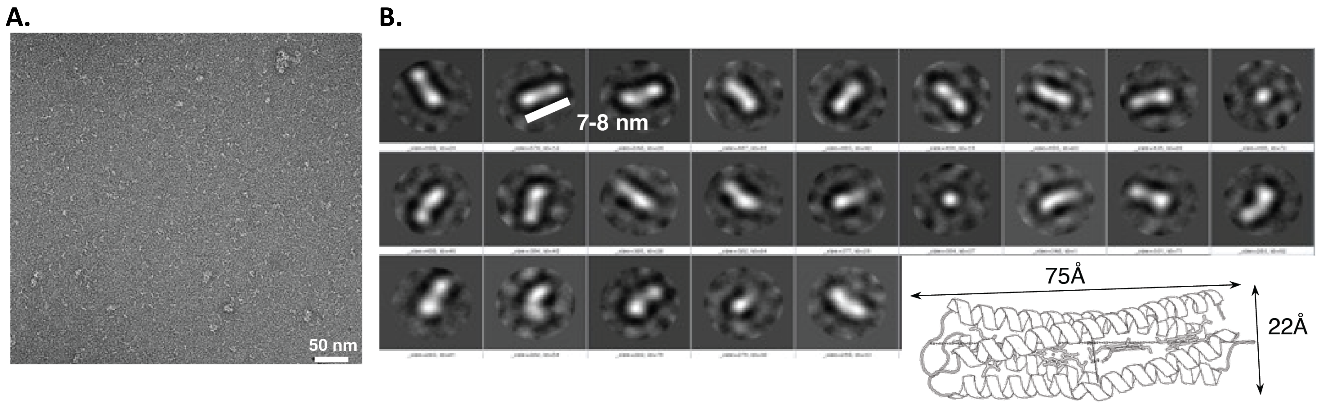

Supplementary Figure 11: Negative stain electron microscopy analysis of e4D2. **A.** Representative image of a micrograph of negatively stained e4D2 (scale bar 50 nm). **B.** Reference-free 2D class averages of e4D2 demonstrate particles consistent with the predicted dimensions of e4D2.

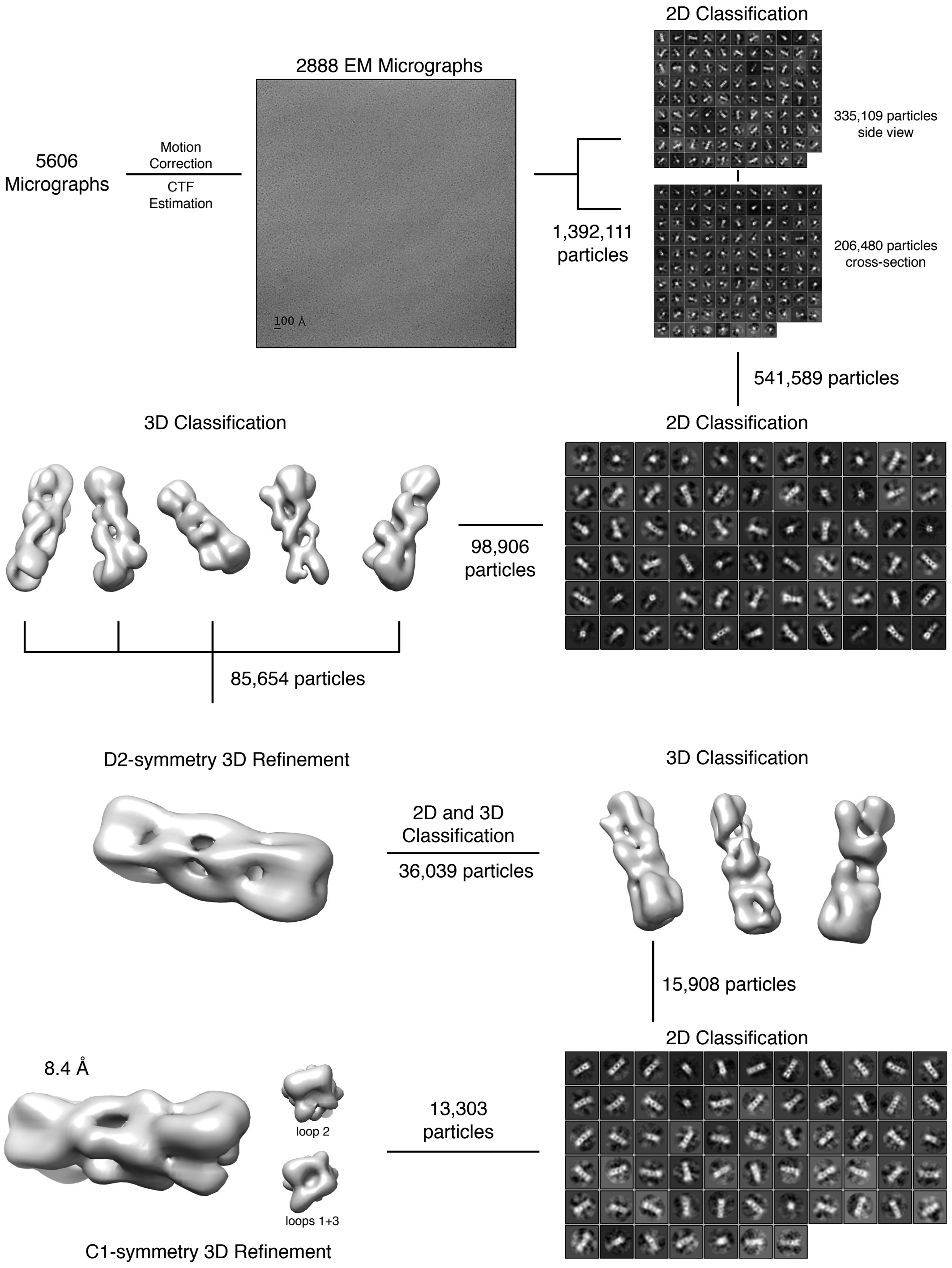

Supplementary Figure 12: Flowchart showing the workflow of e4D2 cryo-EM processing in Relion 3.1. Particles were initially picked based on either a ‘side view’ demonstrating the length of the helices or an ‘end view’ of the helical bundle. These particles were sorted by 2D classification, combined and used for 3D classification. An initial 3D classification and refinement with D2 symmetry highlighted the helical bundle topology. This was followed by 3D classification without any symmetry on a more rigorously filtered particle set utilizing the D2-symmetric model as a reference map, and then 2D classification followed by 3D classification and refinement. This yielded the final map reconstructed from 13,303 particles at a resolution of 8.4 Å which corroborates the helical structure of e4D2 including the asymmetric loop placements.

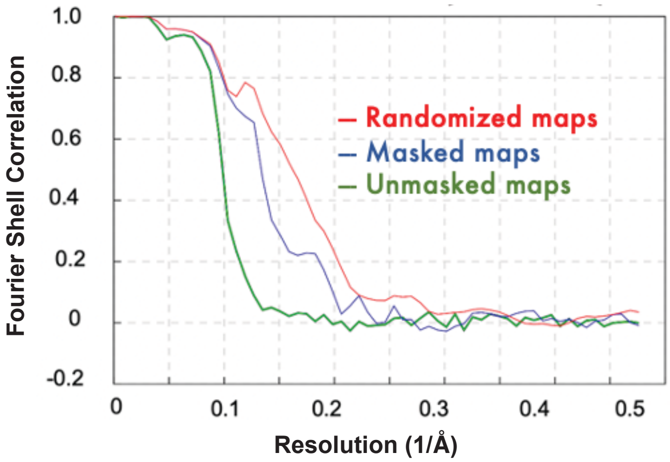

Supplementary Figure 13: Fourier shell correlation (FSC) curve after gold standard refinement for the e4D2 cryo-EM structure indicating a final resolution of 8.4 Å (FSC criterion 0.143).

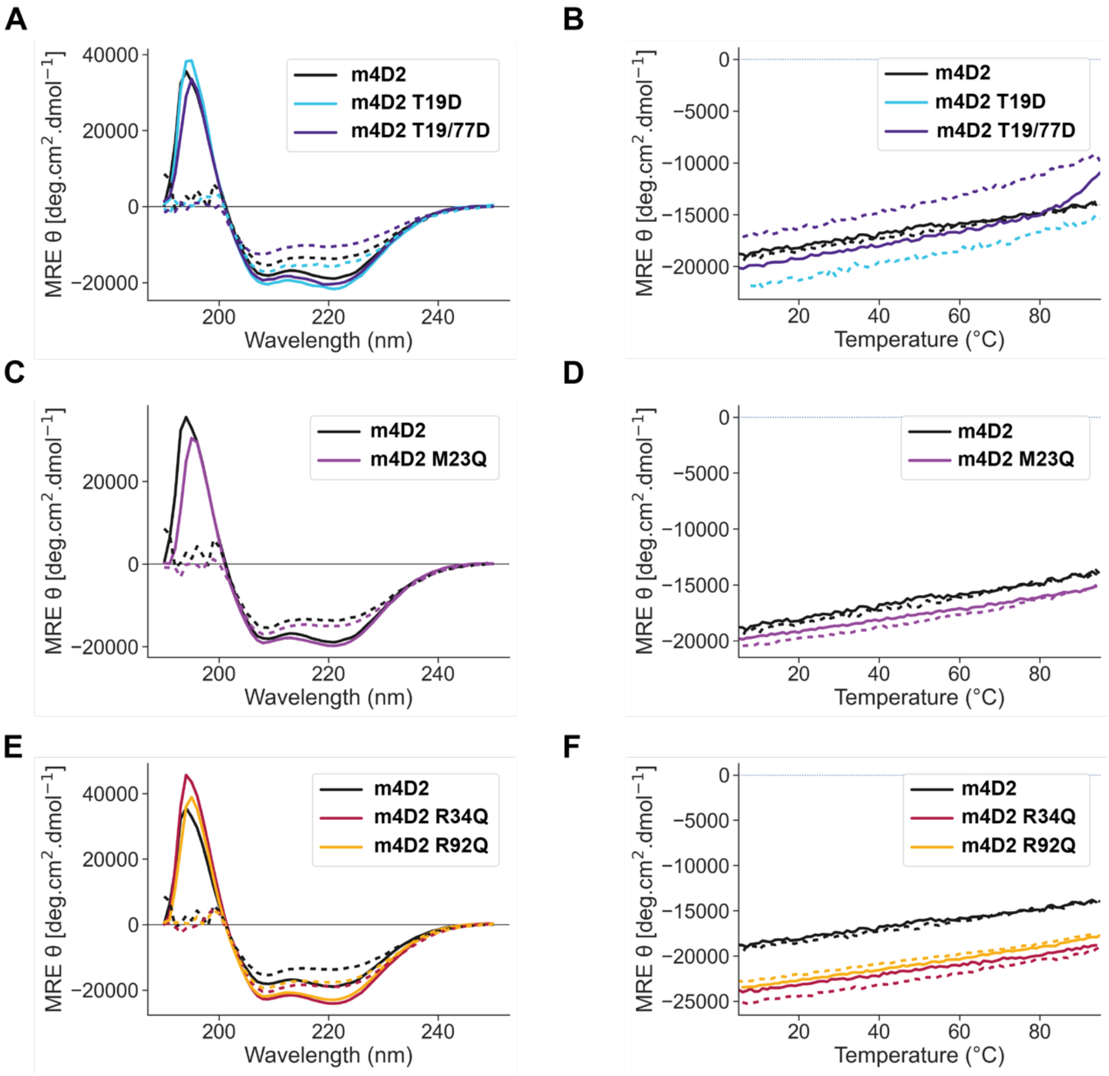

Supplementary Figure 14: Far-UV circular dichroism spectra of heme-loaded m4D2 variants with varying temperature (**A, C, E**). Corresponding temperature dependences of CD signal monitored at 222 nm during denaturation (**B, D, F**). All data was collected in 20 mM CHES, 100 mM KCl, pH 8.6.

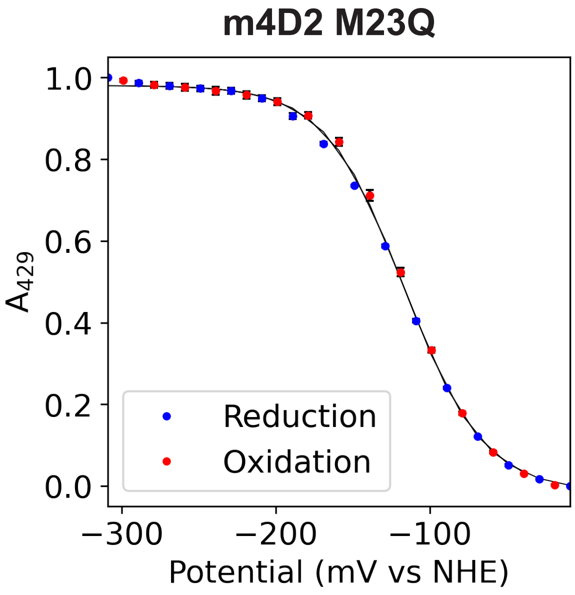

Supplementary Figure 15: Redox potentiometry of m4D2 M23Q recorded in 20 mM CHES, 100 mM KCl, 10% glycerol, pH 8.6. Data were fitted to a single electron Nernst function, indicating a midpoint redox potential of -116 mV vs NHE.

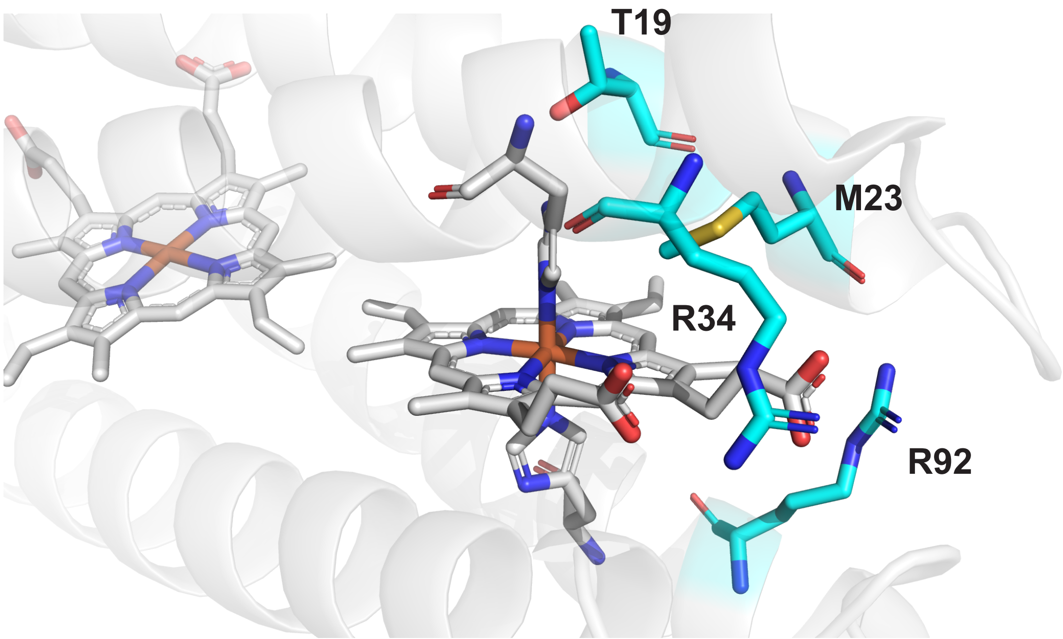

Supplementary Figure 16: Positions for redox altering m4D2 mutations mapped onto the 4D2 crystal structure.

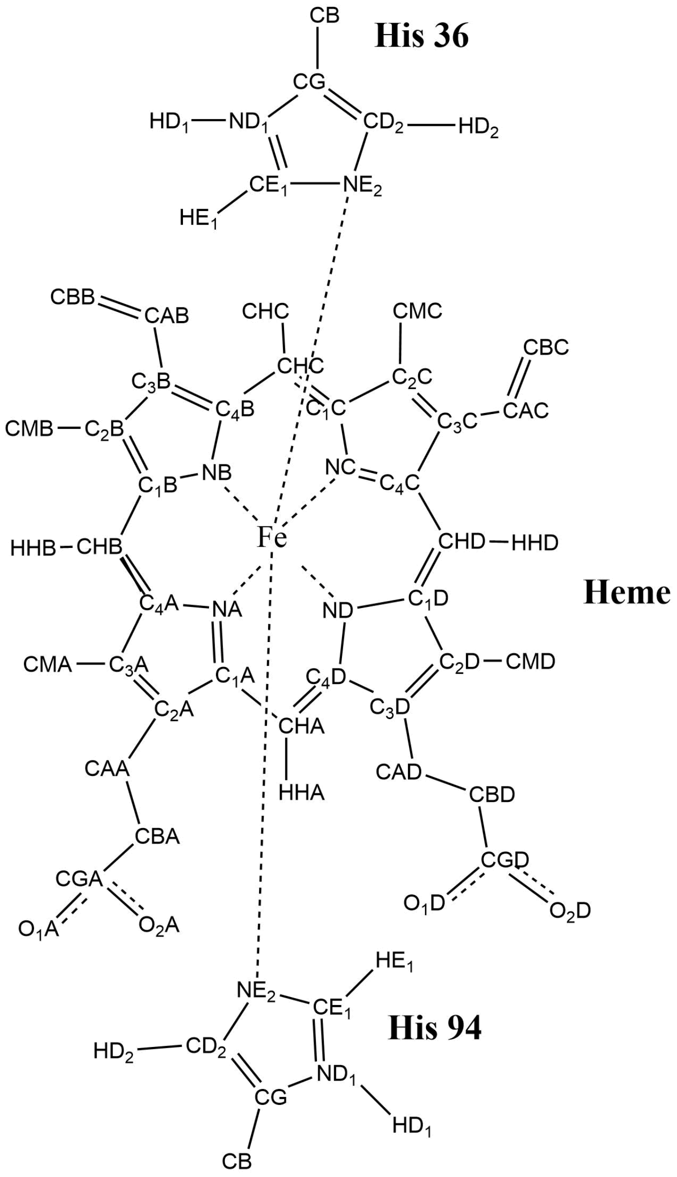

Supplementary Figure 17: Schematic representation of the bis-histidine heme B redox center.

Supplementary Table 1. X-ray data collection and structural refinement statistics

	4D2 (7AH0)	4D2 T19D (8CCR)
**Data Collection**		
Beamline	Diamond Light Source, I03	Diamond Light Source, I04
Space group	H3	H3
Unit cell dimensions (Å): a, b, c	81.25, 81.25, 59.06	81.09, 81.09, 58.89
Unit cell angles (°): a, b, g	90, 90, 120	90, 90, 120
Resolution (Å)	30.24 - 1.91 (1.96 - 1.91)	40.55 – 2.10 (2.16 – 2.10)
R_{merge}	0.129 (1.602)	0.102 (0.367)
Number of reflections	11214 (827)	177549 (14437)
Multiplicity	9.7 (1.81)	21.2 (21.4)
Mean signal to noise ratio (I/σ)	11.1 (1.5)	15.40 (3.80)
Completeness (%)	99.5 (99.4)	100 (100)
**Refinement**		
Resolution (Å)	30.24 - 1.91 (1.96 - 1.91)	40.55 – 2.10 (2.16 – 2.10)
N° unique reflections used in refinement	10644	7531
R_{work}	0.213	0.200
R_{free}	0.219	0.233
N° protein atoms used in refinement	835	849
N° water molecules used in refinement	5	5
Average B-factors (Å^2)	43.0	47.0
R.M.S deviations – length (Å)	0.011	0.022
R.M.S deviations – angle (°)	1.90	2.36
Ramachandran favoured residues (%)	100	100
Ramachandran outlying residues (%)	0	0

Values in parentheses represent the highest resolution shell.

Supplementary Table 2. Cryo-EM data collection and structural refinement statistics. Data were acquired with a 200 kV Talos Arctica (GW4 Facility), GATAN K2 detector and C_s 2.7 mm.

	**e4D2 (EMD-16847)**
**Dose**	
Nominal magnification	130,000x
Pixel Size (Å)	1.05 (0.525)
Pixel Area (Å^2)	1.1025
Dose Rate (e$^-$/Å^2/s)	5.39
Exposure Time (s)	11.5
Total Dose (e$^-$/Å^2)	62.0
Number of Fractions	46
Total dose per Fraction (e$^-$/Å^2)	1.35
**Data Collection & processing**	
Voltage (kV)	200
Defocus Range (µm)	-1.2 to -2.4
Final Particle Images	13,303
Refinement Symmetry	C1
Resolution at FSC = 0.143 (Å)	8.4
Map-sharpening B factor (Å^2)	-262

Supplementary Table 3: Calculated atomic partial charges for bis-histidine ligated heme B.

	**Atomic partial charge**			**Atomic partial charge**	
**Atom name**	**Oxidized**	**Reduced**	**Atom name**	**Oxidized**	**Reduced**
FE	0.894	0.735	C1C	0.038	0.160
NA	-0.035	-0.212	C2C	0.062	0.031
NB	-0.269	-0.317	C3C	-0.271	-0.286
NC	-0.174	-0.367	C4C	0.232	0.318
ND	0.151	0.067	CMC	0.106	0.066
CHA	0.027	-0.089	CAC	-0.117	-0.113
HHA	0.183	0.167	CBC	0.113	0.056
C1A	-0.421	-0.203	CHD	-0.379	-0.448
C2A	0.358	0.175	HHD	0.352	0.341
C3A	-0.301	-0.271	C1D	-0.026	-0.009
C4A	0.021	0.141	C2D	-0.383	-0.290
CMA	0.157	0.101	C3D	0.404	0.233
CAA	0.000	0.000	C4D	-0.462	-0.308
CBA	0.000	0.000	CMD	0.167	0.108
CGA	0.270	0.270	CAD	0.000	0.000
O1A	-0.635	-0.635	CBD	0.000	0.000
O2A	-0.635	-0.635	CGD	0.270	0.270
CHB	-0.042	-0.178	O1D	-0.635	-0.635
HHB	0.125	0.128	O2D	-0.635	-0.635
C1B	-0.327	-0.215	CB	0.146	0.100
C2B	0.229	0.191	CG	0.052	0.086
C3B	-0.573	-0.557	ND1	-0.201	-0.260
C4B	0.830	0.809	HD1	0.336	0.331
CMB	0.108	0.076	CD2	-0.291	-0.352
CAB	-0.036	-0.034	HD2	0.218	0.229
CBB	0.096	0.043	CE1	-0.038	-0.035
CHC	-0.785	-0.833	HE1	0.164	0.159
HHC	0.379	0.364	NE2	-0.102	-0.049

Supplementary Methods

General and molecular biology.

Unless specified, all chemicals were purchased from Merck or Fisher Scientific. The *de novo* heme proteins were cytoplasmically expressed from the pET151 vector (Thermo Fisher) with an N-terminal 6xHis tag and TEV cleavage sequence. Periplasmic expression was utilized to boost non-covalent heme incorporation, and was achieved through expression from a modified pMal-p4x vector (NEB), pSHT[1], retaining the additional N-terminal SEC translocon recognition sequence from the *E. coli* periplasmic maltose binding protein. Artificial gene sequences were synthesized by Eurofins Genomics and cloned by a Site-Directed Ligase Independent Cloning method, using Q5 polymerase (NEB) to amplify gene and vector sequences prior to hybridization of overhanging fragments. Cloned vectors were transformed into Stellar cells (Takara) prior to plasmid purification at a concentration of 30-100 ng/µL using a plasmid DNA miniprep kit (NEB).

Protein expression and purification.

All proteins were expressed in the T7 Express *E. coli* strain (NEB). Transformed colonies were cultured overnight in 100 mL LB with carbenicillin at a concentration of 50 µg/mL, and 20 ml of culture were transferred to 1 L of LB which was grown at 37°C to an OD_{600nm} of 0.6 prior to induction by addition of 1 mM IPTG. Proteins were expressed for four hours at 37°C, after which cells were harvested by centrifugation at 4000 xg for 25 minutes and resuspended in lysis buffer (50 mM sodium phosphate, 300 mM sodium chloride, 20 mM imidazole, pH 8).

Resuspended cell-pellets were lysed by four 20-second sonication pulses on ice, and cell debris was removed by centrifugation at 18,000 xg. Designs were initially purified from lysate by nickel affinity chromatography (HisTrap column, GE), eluting at an imidazole concentration up to 250 mM. The proteins were immediately dialyzed overnight (SnakeSkin 3.5 kDa MWCO, Thermo Fisher) into a low salt buffer for TEV cleavage (50 mM Tris, 0.5 mM EDTA, pH 8), which was initiated through the addition of tris(2-carboxyethyl)phosphine hydrochloride (TCEP) to 1 mM and approximately 200 µg of TEV protease per 3 L of original culture media. Cleaved designs were further purified by nickel affinity chromatography, followed by size exclusion chromatography (GE HiLoad 16/600 Superdex 75pg) under final buffer conditions of 20 mM CHES, 50 mM potassium chloride at pH 8.6. Purified protein samples were concentrated using centrifugal concentrators with a molecular weight cut-off of 3 kDa (Vivaspin).

If necessary, exogenous heme was added *in vitro* to fully saturate cofactor binding sites by dropwise addition of hemin dissolved at 1 mg/mL in DMSO to a modest (i.e. 1.5-fold) excess. Excess heme was removed by gel filtration using G25 desalting columns or a further round of size exclusion chromatography. Apo samples were prepared by acidic 2-butanone extraction of the heme cofactor[2] followed by exhaustive dialysis to remove the organic solvent, then size exclusion chromatography in 20 mM CHES, 50 mM potassium chloride at pH 8.6.

Porphyrin binding titrations.

Cofactor binding titrations were carried out by preparation of up to 1 mL of 2-3 µM apo protein solution in a quartz cuvette, followed by incremental addition of porphyrin in small aliquots of DMSO (0.5-2 µL). Full absorbance spectra between 200-800 nm were measured after each addition and following thorough mixing. Heme dissociation constants (*K_D*) were calculated by fitting absorbance at a single wavelength to the quadratic tight binding equation (Eq. 1):

$$Absorbance = [C] \times \varepsilon_{free} + (\varepsilon_{bound} - \varepsilon_{free}) \frac{K_D + [P_{tot}] + [C] - \sqrt{(K_D + [P_{tot}] + [C])^2 - 4[P_{tot}][C]}}{2} \quad (1)$$

$[C] =$ Cofactor Concentration (µM)
$\varepsilon_{free} =$ Unbound Cofactor Extinction Coefficient ($\mu M^{-1} cm^{-1}$)
$\varepsilon_{bound} =$ Bound Cofactor Extinction Coefficient ($\mu M^{-1} cm^{-1}$)
$[P_{tot}] =$ Total Protein Concentration (µM)

Mass spectrometry.

The molecular weight of heme-bound protein complexes was measured using electrospray ionization-mass spectrometry (ESI-MS). Data was acquired using a Waters Xevo G2-XS QTof LC-MS instrument, bypassing the liquid chromatography system by direct injection of purified protein samples at concentrations of 50-200 µM prepared in an aqueous 100 mM ammonium acetate solution. The sample was injected at a flow rate of 0.25 mL/minute of ammonium acetate, with a capillary voltage of 3.6 kV and cone voltage of 50 V, in positive ion mode. Predicted ion m/z values were calculated by the following equation (Eq. 2), assuming the ionization occurred by gaining a proton, increasing the mass (m) by 1 g/mol per charge (Z).

$$m/z = \frac{m+Z}{Z} \quad (2)$$

The molecular weight of the apo proteins was validated by Matrix-assisted laser desorption/ionization (MALDI) MS, acquired on Bruker UltrafleXtreme spectrometer. Protein samples (50 µM) were dissolved 1:10 in a matrix solution of 10 mg/mL sinapinic acid in 50:50 water/acetonitrile with 0.1% trifluoroacetic acid.

Redox potentiometry.

Heme redox potentials were measured by OTTLE potentiometry (Optically transparent thin-layer electrochemistry) as described previously[3]. All designed proteins were prepared at a concentration of 50 µM in 10% glycerol, 20 mM CHES, 100 mM KCl at pH 8.6. A total of six redox mediators were added at the following concentrations: phenazine ethosulfate 20 µM, indigotrisulfonate 50 µM, duroquinone 6 µM, 2-hydroxy-1,4-napthoquinone 25 µM, phenazine 20 µM, anthroquinone-2-sulfonate 20 µM. UV-visible spectra were measured using a Cary 60 spectrophotometer whilst a potential was applied across a custom built quartz OTTLE cell by a

Biologic SP-150 potentiostat using a thin platinum gauze working electrode, a platinum counter electrode, and an Ag/AgCl reference electrode (RE-5B, BASi). Potentials were corrected to mV vs the Nernst hydrogen electrode (NHE) through calibration with cytochrome *c*, resulting in an average adjustment of +220 mV. Midpoint potentials (E_m) were derived by fitting oxidation and reduction data to the following one (Eq. 3) or two (sequential) (Eq. 4) electron Nernst equations, where mV is the applied potential and Abs is the absorbance associated with the ferrous Soret peak (approximately 429 nm).

$$1e^- \qquad Abs = \left(A + B.10^{\frac{mV - E_m}{59}} \right) / \left(1 + 10^{\frac{mV - E_m}{59}} \right) \tag{3}$$

$$2 \times 1e^- \qquad Abs = \left(A.10^{\frac{mV - E_{m1}}{59}} + C + B.10^{\frac{mV - E_{m2}}{59}} \right) / \left(1 + 10^{\frac{mV - E_{m1}}{59}} + 10^{\frac{mV - E_{m2}}{59}} \right) \tag{4}$$

Circular dichroism spectroscopy.

Circular dichroism (CD) spectra were recorded using a JASCO J-1500 spectrophotometer. All protein samples were prepared at a concentration of approximately 5-15 µM, buffer solution (CHES 20 mM, KCl 100 mM, pH 8.6) was used to provide a baseline for the protein spectra. The CD signal was measured at 222 nm whilst raising or lowering temperature between 5-95°C, at a rate of 1°C per minute, to track the overall helicity of designs. Full CD spectra were measured between 190-250 nm at specific temperature points, acquiring eight spectra at each condition and averaging the results. The raw circular dichroism data was converted to mean residual ellipticity (MRE) using the following equation (where n is the number of peptide bonds in a protein sample) (Eq. 5):

$$MRE\ (deg.cm^2.dmol^{-1}) = \frac{Raw\ Elipticity\ (mdeg) \times 10^6}{Path\ Length\ (mm) \times Protein\ Concentration\ (\mu M) \times n} \tag{5}$$

References

1 Anderson, J. L. R. *et al.* Constructing a man-made c-type cytochrome maquette in vivo: electron transfer, oxygen transport and conversion to a photoactive light harvesting maquette. *Chem Sci* **5**, 507-514 (2014). https://doi.org:10.1039/C3SC52019F

2 Teale, F. W. Cleavage of the haem-protein link by acid methylethylketone. *Biochim Biophys Acta* **35**, 543 (1959). https://doi.org:10.1016/0006-3002(59)90407-x

3 Ost, T. W. *et al.* 4-cyanopyridine, a versatile spectroscopic probe for cytochrome P450 BM3. *J Biol Chem* **279**, 48876-48882 (2004). https://doi.org:10.1074/jbc.M408601200
